# Nature exposure reduces self-reported pain: a systematic review and meta-analysis

**DOI:** 10.1038/s44220-025-00569-2

**Published:** 2026-01-06

**Authors:** Maximilian Oscar Steininger, Jonas Paul Nitschke, Mathew Philip White, Claus Lamm

**Affiliations:** 1 Social, Cognitive, and Affective Neuroscience Unit, Department of Cognition, Emotion, and Methods in Psychology, Faculty of Psychology, University of Vienna, Vienna, Austria; 2 Department of Clinical and Health Psychology, Faculty of Psychology, University of Vienna, Vienna, Austria; 3 Cognitive Science Hub, University of Vienna, Vienna, Austria; 4 European Centre for Environment and Human Health, University of Exeter, Truro, UK; 5 Environment and Climate Research Hub, University of Vienna, Vienna, Austria

**Keywords:** Psychology, Quality of life

## Abstract

Pain is a global health issue with substantial individual, societal and economic impacts. Given the risks of pharmacological treatments, complementary approaches to pain management are essential. Nature exposure has emerged as a promising nonpharmacological strategy, but evidence of its effectiveness is inconclusive. Here in this systematic review and meta-analysis we examined 62 studies (96 effects) across 21 countries, including 4,439 participants, to assess the impact of nature exposure on self-reported pain. The results indicate a significant small-to-moderate reduction in pain associated with nature exposure (standardized mean difference of 0.53), but studies exhibited moderate-to-high risk of bias and substantial heterogeneity. Studies evaluating nature against matched comparators reported effects roughly half the size of those using nonmatched controls and multisensory stimuli tended to show stronger effects. These findings support nature as a promising complementary pain management strategy. However, high heterogeneity and risk of bias warrant caution and highlight the need for more rigorous research.

## Abstract (web summary)

The authors conducted a systematic review and meta-analysis of 62 studies, including more than 4,400 participants across 21 countries, to investigate the effects of nature exposure on self-reported pain.

## Main

Pain is a major global health issue, with more than a fifth of adults experiencing pain regularly or chronically^1–3^. This burden not only affects individuals, contributing to disabilities and impairing daily life activities, but also imposes substantial costs on societies and economies. Treatments associated with back pain alone account for approximately 1.5% of Europe’s gross domestic product annually, while chronic pain is the single most expensive medical issue^4–6^. Moreover, pain and mental disorders often co-occur^7^ and more pain is associated with more severe mental health symptoms^8^, exacerbating the negative impact of pain. Consequently, effective treatments are needed to alleviate individual and socioeconomic burdens and costs. However, the complex and multifaceted nature of pain represents a major challenge to identifying such treatments.

Pain treatment often involves multimodal approaches rooted in biopsychosocial frameworks, integrating pharmacological and nonpharmacological strategies^9,10^. While pharmacological treatments, such as opioid and nonopioid medications, are well-established and effective^11^, they carry substantial risks, including side effects, tolerance and the potential for addiction^12,13^. Furthermore, given the biopsychological nature of pain, integrative approaches—including psychological (for example, cognitive behavioral therapy), physiological (for example, massage) and complementary treatments (for example, acupuncture)—play an important role alongside pharmacological interventions^9^. While these treatments are widely adopted by individuals experiencing pain^14^, there is a growing need to systematically evaluate the effectiveness of these adjunct strategies. One increasingly recognized adjunct strategy is nature exposure, which has been repeatedly linked to analgesic effects^15,16^.

Previous studies have shown that nature exposure is associated with reduced self-reported acute or chronic pain, with several pathways proposed to explain how nature may achieve this effect^17^. For one, factors indirectly facilitated by contact with nature might be important. For example, nature contact promotes physical activity and social integration^18^, both known to positively affect pain regulation^19,20^. For another, exposure to specific natural elements, such as volatile organic compounds and environmental microbiota, may also influence pain. This may explain why residing near greenspaces and rural areas is associated with healthier microbiome signatures^21^ that are, in turn, linked to improved pain outcomes^22^. While several of these pathways have been predominantly explored in chronic pain, evidence suggests that acute pain induced by medical or experimental procedures can also be alleviated by nature. Notably, in such settings, mere exposure to nature sights and sounds may be sufficient to reduce self-reported pain.

Consequently, several studies have investigated whether nature stimuli alone can reduce pain, but results are mixed, leaving the evidence inconclusive. In a seminal study, Ulrich^16^ showed that following painful surgery, patients with a room offering a view of greenspace required lower doses of analgesics compared to those who could only see a brick wall from their hospital bed. Subsequent studies across various contexts involving experimental or medical procedures that induced acute pain have supported this finding. For example, research has shown that viewing or listening to nature can reduce acute self-reported pain during procedures such as flexible bronchoscopy^23^, colonoscopy^24,25^, dental treatments^15^, burn wound dressings^26,27^ or experimentally induced pain^28^. However, the reported effects vary widely, with some studies indicating no effects^29,30^ and others showing detrimental outcomes (that is, an increase in pain)^31,32^.

Variations and inconsistencies in research designs probably contributed to these inconclusive findings as studies differed in methodological choices. For instance, studies varied widely in their selection of comparators. As with other complementary pain management approaches, the literature features diverse comparator conditions and is marked by methodological shortcomings^14^. A common approach is to compare nature interventions to no alternative stimulation or treatment as usual (TAU)^33^, introducing several challenges. Comparisons with no stimulation complicate isolating the intervention’s specificity as they are influenced by both its impact and placebo or expectancy effects^34^. In addition, such comparisons do not clarify whether the effect is uniquely attributable to nature or stems from generic aspects shared with other environments or stimuli. While using TAU as a comparator is clinically relevant, it can hinder cross-study comparisons as TAUs may vary depending on the medical procedure or institution^35^. Furthermore, the studies differed in the types of nature stimuli used. Some studies used simple, unimodal stimuli, such as listening to nature sounds^36^ or viewing still images of nature^37^, while others employed multimodal, highly interactive interventions^38^, which may more effectively reduce pain. Thus, understanding the relative effectiveness of nature, considering both the conditions it is compared against and the specifics of its implementation, is crucial for guiding practitioners and researchers toward its optimal application. Identifying the contexts and factors that maximize the effectiveness of nature exposure underscores the need for a thorough investigation into the underlying causes of outcome variations.

Therefore, although nature exposure has garnered significant attention as a potential adjunct analgesic method, its effectiveness in reducing self-reported pain remains underspecified and inconsistently explored. Existing studies appear to vary widely in study design, methodological characteristics and overall quality, yet the extent and implications of this variability have not been systematically assessed. To address these gaps, we conducted a preregistered systematic review and meta-analysis to quantitatively integrate the available evidence. We exclusively included studies investigating the effects of nature exposure on acute or spontaneously occurring chronic pain. Our aims were to estimate the overall effect of nature exposure on pain, assess its robustness and generalizability, evaluate methodological quality and potential bias and examine how design and contextual factors contribute to variability in findings. Specifically, we hypothesized that nature exposure (versus non-nature controls) would significantly reduce self-reported acute or spontaneously occurring chronic pain, and preregistered several potential moderators related to study design and methodological features. By systematically coding and analyzing these features, we aimed not only to clarify the evidence base but also to critically reflect on current research practices—ultimately contributing to the development of more rigorous and methodologically sound studies in this emerging interdisciplinary research domain^39^.

## Results

### Study characteristics

**Fig. 1:**
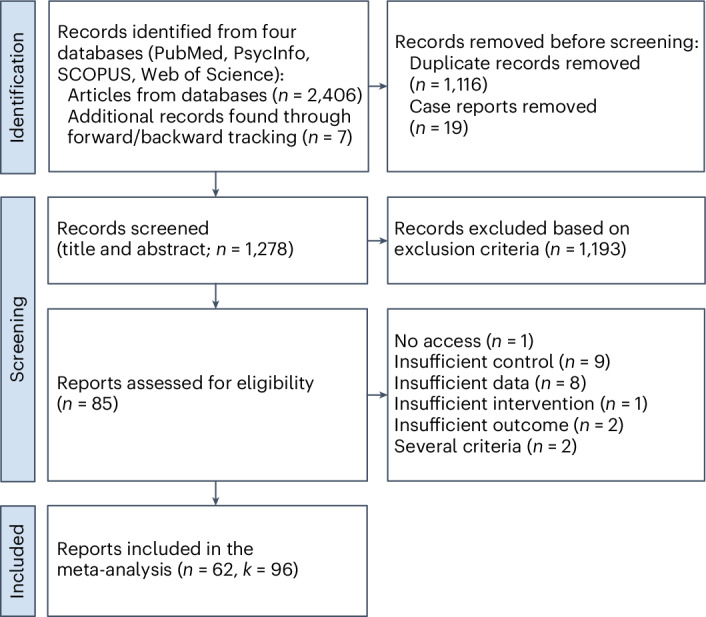
PRISMA flow chart for the systematic review and meta-analysis. Inclusion criteria were as follows: (1) adult participants from healthy or clinical populations, (2) exposure to a painful medical or experimental procedure, (3) at least one primarily natural stimulus targeting one or more sensory modalities, (4) at least one control group or condition involving exposure to a non-natural stimulus, (5) measurement of self-reported pain, (6) articles published in English and (7) peer-reviewed articles containing original research (that is, no reviews or opinion pieces).

Figure 1 summarizes the selection of studies and the applied exclusion criteria for each step in a Preferred Reporting Items for Systematic reviews and Meta-Analyses (PRISMA) flow diagram. After the initial screening of the abstracts and titles, we identified 85 potentially relevant studies. Of these, 23 studies were further excluded following inspection of their precise content using the full texts (Supplementary Table 1). The final sample included *n* = 62 studies encompassing *k* = 96 extracted effects and a total sample size of *N* = 4,439 participants, ranging from *n* = 8 to *n* = 270 participants per study. Table 1 summarizes the included studies, reporting effect sizes per study, a brief description of the pain procedure, its context and measurement as well as the nature and control conditions. A comprehensive overview, including full references, detailed effect size information, study design and context, sample sizes, pain types and measurements, detailed descriptions of nature and control conditions (with moderator coding for purity, immersiveness, interactivity and type of control) and sources of extracted data for each study, is provided in Supplementary Table 2. The studies were published between 1992 and 2024 and conducted across 21 different countries, including Brazil (1; 1.6%), China (5; 7.7%), Denmark (2; 3.2%), France (1; 1.6%), Germany (1; 1.6%), Hong Kong (2; 3.2%), Iran (6; 9.7%), Israel (2; 3.2%), Italy (1; 1.6%), Japan (1; 1.6%), Jordan (1; 1.6%), Malaysia (1; 1.6%), the Netherlands (3; 4.8%), Poland (2; 3.2%), Spain (2; 3.2%), Sweden (1; 1.6%), Switzerland (1; 1.6%), Thailand (1; 1.6%), Turkey (8; 12.9%), the UK (3; 4.8%) and the USA (17; 27.4%).

**Table 1:** Studies included in the multilevel meta-analysis (with key characteristics)

Ref.	*k*	Pain (context; outcome)	Nature intervention	Comparator
^27^	1	Wound care (med; NRS)	Nature scene (2D) + music	TAU
^64^	2	Sigmoidoscopy (med; VAS)	1: Nature sounds; 2: Nature scene (2D HMD) and sounds	TAU
^139^	2	Ischemic (exp; 1: Thr; 2: Tol)	Nature scene (2D HMD)	Black screen
^140^	2	Ischemic (exp; 1: Thr; 2: Tol)	Nature scene (2D HMD)	Black screen
^23^	1	Bronchoscopy (med; NRS)	Nature mural and sounds	TAU
^141^	1	Thermal (exp; GRS)	Icy canyon (VR) + interactive elements (SnowWorld)	Fixation cross
^24^	2	Colonoscopy (med; VAS)	1: Nature scene (2D HMD) + music; 2: Nature scene	TAU
^142^	1	Thermal (exp; GRS)	Icy canyon (VR) + interactive elements (SnowWorld)	Fixation cross
^97^	2	Dental (med; VAS)	Interactive botanical garden (VR)	1: Movie; 2: Sham HMD
^32^	1	Nerve block (med; NRS)	Nature scene (2D) and sounds + music	TAU
^54^	2	Bone marrow biopsy (med; VAS)	Nature mural and sounds	1: TAU; 2: Urban mural and sounds
^26^	1	Wound care (med; GRS)	Icy canyon (VR) + interactive elements (SnowWorld)	TAU
^36^	2	Thermal (exp; VAS)	Nature sounds: 1 (rain); 2 (water)	Pink noise
^99^	2	Laser-evoked potential (exp; VAS)	VR nature waiting room: 1 (chronic); 2 (healthy)	VR standard waiting room
^94^	1	Mechanical ventilation (med; VAS)	Nature sounds	Sham headphones
^88^	2	Wound care (med; VAS)	Nature scenes and sounds: 1 (2D); 2 (VR)	Not specified
^52^	1	Wound care (med; NRS)	Icy canyon (VR) + interactive elements (SnowWorld)	TAU
^65^	2	Chemotherapy (med; VAS)	Nature sounds	1: Affirmative sentences; 2: TAU
^66^	1	Cancer-related pain (med; VAS)	Nature scene (window)	Urban scene (window)
^37^	2	Surgery (med; VAS)	Nature scene (2D)	1: Music; 2: TAU
^143^	1	Electric (exp; VAS)	Nature scene (2D)	Black screen
^144^	1	Burn wounds (exp; GRS)	Icy canyon (VR) + interactive elements (SnowWorld)	TAU
^15^	4	1–2: Thermal (exp; NRS); 3–4: Dental (med; NRS)	Nature scene (VR): 2 (passive), 1, 3 and 4 (interactive)	1–3: Sham HMD; 4: Urban scene (interactive VR)
^145^	2	Cesarean section (med; VAS)	Nature sounds	1: Sham headphones; 2: TAU
^40^	1	Chronic pain (med; VAS)	Nature scene (VR) and sounds + music and guided meditation	TAU
^28^	2	Thermal (exp; 1: Thr; 2: Tol)	Nature scene (VR) and sounds + music	Opera scene (VR) + music
^146^	2	Thermal (exp; Tol)	Interactive nature scene (VR)	1: Interactive VR; 2: Black screen
^147^	2	Electrical (exp; NRS)	Nature scene (VR) and sounds: 1 (interactive); 2 (passive)	Black screen
^148^	1	Wound care (med; VAS)	Nature scene (VR) and sounds	TAU
^149^	2	Intramuscular injection (med; VAS)	Nature scene (not specified)	1: Optical illusions; 2: TAU
^80^	1	Colonoscopy (med; VAS)	Nature scene (VR) and sounds + music	TAU
^67^	1	Hysteroscopy (med; NRS)	Nature scene (VR) + narration	TAU
^29^	2	Vasectomy (med; VAS)	Nature scene: 1 (2D HMD); 2 (VR)	TAU
^68^	1	Breast biopsy (med; VAS)	Nature scene (VR) and sounds + music	TAU
^81^	4	Electric (exp; 1 and 3: Thr, 2 and 4: Tol)	Nature scene: 1–2 (2D); 3–4 (in situ; green)	Black screen
^93^	1	Cystoscopy (med; NRS)	Nature scene (VR) and sounds	TAU
^82^	1	Amniocentesis (med; VAS)	Nature scene (not specified)	TAU
^69^	1	Labor contractions (med; NRS)	Nature scene (VR) and sounds	TAU
^30^	2	Chemotherapy (med; NRS)	Nature scene: 1 (window and mural; green); 2 (interactive VR)	TAU
^91^	1	Hysteroscopy (med; NRS)	Nature scene (VR) and sounds + music	TAU
^96^	2	Prostate biopsy (med; VAS)	Nature scene (VR)	1: Stress ball; 2: TAU
^70^	1	Colonoscopy (med; VAS)	Nature scene (VR) + music	TAU
^25^	1	Colonoscopy (med; VAS)	Nature scene (not specified) + music	Sham HMD
^76^	1	Intravenous catheter (med; VAS)	Interactive nature scene (VR)	Sham HMD
^95^	1	Surgery (med; NRS)	Nature scene (not specified) + music	TAU
^71^	2	Surgery (med; VAS)	Nature scene (VR)	1: Educational VR; 2: TAU
^100^	1	Thermal (exp; NRS)	Nature scene (VR) and sounds + guided respiration	Not specified
^83^	3	Colonoscopy (med; VAS)	Nature scene (VR) and sounds + music	1: Stress ball; 2: Music; 3: TAU
^98^	1	Surgery (med; NRS)	Nature scene (2D HMD) + narration and music	Music
^72^	2	Knee arthroplasty (med; VAS)	Interactive nature scene + biofeedback: 1 (2D); 2 (VR)	TAU
^77^	1	Endovascular procedure (med; NRS)	Interactive nature scene (VR) + respiration and hypnosis	TAU
^87^	4	Thermal (exp; NRS)	Nature scene and sounds + respiration: 1 and 3 (2D); 2 and 4 (VR)	Fixation cross
^31^	2	Traumatic injury (med; NRS)	Nature scene + music: 1 (2D); 2 (VR)	Sham HMD
^92^	1	Hysteroscopy (med; VAS)	Interactive nature scene (VR) + music	TAU
^150^	1	Prostate biopsy (med; VAS)	Nature scenes (VR) + music	TAU
^73^	1	Hysteroscopy (med; NRS)	Interactive nature scene (VR) + music and respiration	TAU
^74^	1	Various stimuli (med; NRS)	Nature scene (not specified)	TAU
^78^	1	Bronchoscopy (med; VAS)	Nature scene (VR) + music	TAU
^75^	2	Electrical (exp; Thr)	Nature scene (2D HMD) + music	1: Video game; 2: Not specified (TAU)
^84^	1	Colonoscopy (med; VAS)	Nature scene (VR) + music	TAU
^33^	1	Breast biopsy (med; VAS)	Nature scene (VR) and sounds	TAU
^38^	1	Chronic pain (med; NRS)	Interact with natural materials	Interact with synthetic materials

For studies including more than one effect, the conditions are indicated by numbers within corresponding rows. Exp, experimental; *k*, number of effects; med, medical; thr, threshold; tol, tolerance.

In terms of study design, 26 studies (41.9%) utilized a between-participant design, 17 studies (27.4%) employed a within-participant design and the remaining 19 studies (30.6%) adopted a pre–post control group design. Most studies (47; 75.8%) were conducted in medical settings, while the remaining studies (15; 24.2%) were conducted in experimental settings (these two categories were coded for moderator analyses as context). Notably, one study included two separate experiments, one in a medical setting and the other in an experimental setting^15^. Studies conducted in medical settings were characterized by a diverse range of pain induction procedures, ranging from relatively minor (for example, blood draws) to moderate (for example, colonoscopy) and major (for example, burn wound dressing) medical procedures. Notably, two studies conducted in the medical setting assessed chronic pain that occurred spontaneously rather than being induced by a specific procedure^38,40^. In experimental settings, pain was induced through heat, cold, electrical or ischemic pain. The pain was measured in 32 cases (51.6%) using ratings on a visual analog scale (VAS), in 20 cases (32.3%) on a numerical rating scale (NRS) and in 4 cases (6.4%) on a graphical rating scale (GRS). In the remaining 6 cases (9.7%) pain threshold and/or tolerance measures were used. All results based on ratings assessed the intensity of the painful experience, that is, the perceived strength of the sensation rather than emotional or motivational reactions.

Our inclusion criterion required studies to feature interventions involving one or more stimuli primarily using natural elements (Methods). As a result, the included nature interventions used in the studies covered a broad spectrum of stimuli. These ranged from relatively simple stimuli, such as murals or still images of natural environments or nature soundscapes (for example, waterscapes, birds or forest sounds), to more complex multimodal stimuli. Complex stimuli included images or videos with accompanying soundscapes, virtual reality (VR) environments that facilitated active-navigation and interaction using controller devices, or real-life (in situ) exposure, where participants were seated in outdoor greenspaces or interacted with biotic (for example, plants) and abiotic elements (for example, soil). Out of 96 extracted effects, 48 (50%) involved pure nature interventions characterized solely by natural stimuli, while the remaining 48 (50%) involved nature interventions that also included non-natural, potentially confounding elements, including calming music, video game aspects (for example, target shooting), guided meditation, autohypnosis, breathing (respiration) exercises or narration. Immersiveness corresponded to the number of sensory modalities stimulated by the intervention (for example, an intervention with nature sights and sounds was coded as ‘two’). Thirty-three (33.4%) of the nature interventions were manually coded for our analyses with an immersiveness level of one (mainly vision only), 44 (45.8%) with a level of two, 16 (16.7%) with a level of three and the remaining three (3.1%) effects with a level of four. All effects categorized as level ‘four’ corresponded to interventions conducted in real outdoor settings. In these cases, based on the information provided in the papers, we assumed that participants could see, hear, smell and touch their surroundings. Regarding the interactivity of the nature interventions, we coded 31 (32.3%) effects as passive-attending (observing the nature stimulus without the possibility for interaction), 24 (25%) effects as active-attending (exploring the stimulus via head movements in 360° presentations of static images or videos), 11 (11.5%) effects as active-navigation (navigating the stimulus for example, with VR controllers) and 24 (25%) effects as active-manipulation (direct interaction or manipulation of the stimulus). Six effects (6.3%) could not be coded owing to incomplete information and were treated as having missing data.

Comparators varied widely across studies. We coded comparators (72; 75%) as either nonmatched (for example, TAU) or relatively matched (21; 21.9%). Three effects (3.1%) could not be coded due to insufficient information in the articles. Nonmatched comparators included TAU, viewing a black computer screen or a fixation cross, or wearing (deactivated) devices, such as turned-off headphones or head-mounted displays (HMD), to control for delivery effects. Matched comparators had notable variations in how closely they were matched to the nature interventions. Some were closely matched (for example, images of nature scenes versus images of urban scenes or natural biotic and abiotic elements versus their synthetic imitations). Others targeted the same sensory modalities but presented less well-matched content (for example, comparing the soundscape of a beach with audio recordings of affirmative sentences). The remaining comparators were only loosely matched in content or sensory modalities engaged (for example, a 360° VR scene of a beach walk compared to squeezing a stress ball).

We used the revised Cochrane risk of bias tool^41^, a standardized framework that evaluates bias across five domains (Methods). Each study received domain-specific and overall ratings of ‘low’, ‘some concerns’ or ‘high’ risk of bias, based on responses to predefined signaling questions. Using the tool, 33 studies (53.2%) were classified as having a ‘high’ overall risk of bias, while the remaining 29 (46.8%) were coded as having at least ‘some concerns’. The most frequent ratings of ‘some’/‘high’ concerns were observed in categories D4 (measurement of the outcome; 96.7%), D1 (randomization process; 66.1%) and D5 (selection of reported result; 50%). The almost universal category D4 concerns reflect that the studies in this review focused on self-reported pain and participants were typically aware of the intervention they received (or that they received an intervention, compared to TAU). For D5, most concerns were related to study protocols not being registered (32.2%). Categories D2 (deviations from intended interventions) and D3 (missing outcome data) generally exhibited low concern across studies (95.2% and 90.3%, respectively).

In summary, the included studies employed different research designs, were predominantly conducted in medical settings and primarily assessed pain using VAS or NRS. There was considerable heterogeneity in the nature interventions, comparators and types of pain assessed. The pain varied from minor to major interventions or procedures and most studies compared nature to nonmatched comparators. Notably, half of the effects examining nature interventions included confounding factors and most interventions were characterized by low-to-moderate levels of immersion or interactivity. Last, primarily owing to the subjective nature of the outcome variable, the overall concern for risk of bias was rated as moderate-to-high across all studies. However, this concern largely reflects limitations inherent to the research question, such as the subjectivity of the outcome variable or the difficulty of blinding participants to nature interventions, rather than avoidable flaws in design, execution, or interpretation.

### Effects of nature exposure on self-reported pain

**Fig. 2:**
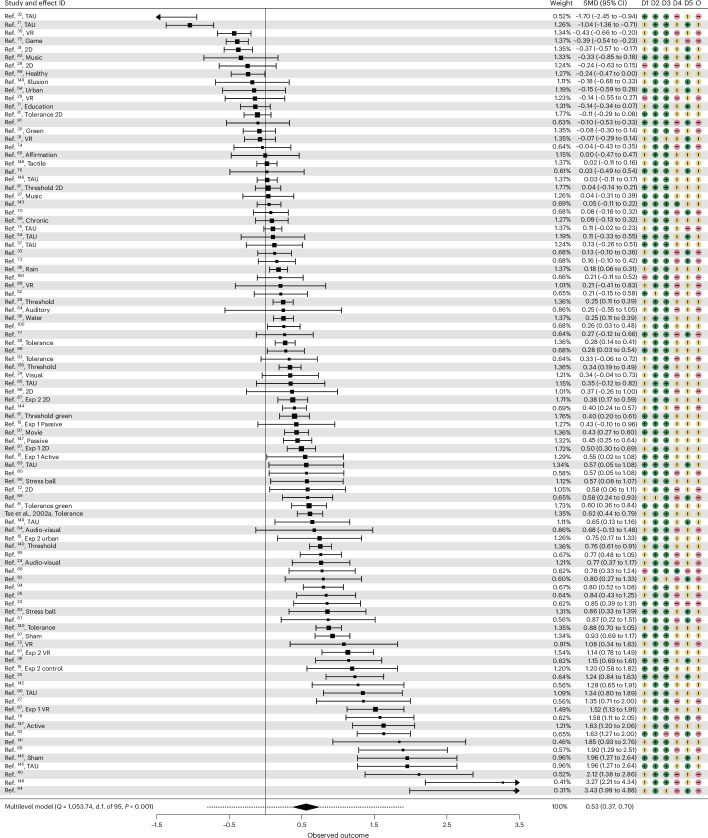
Forest plot depicting the effect of nature interventions on self-reported pain using individual effect sizes. For a plot using within-study aggregated estimates, see Supplementary Fig. 1. The descriptions for effect IDs indicate the individual effects for studies with multiple effect sizes (Table 1). Individual effect sizes (SMD) are shown as squares, with error bars indicating the 95% CIs. Higher and positive values represent reductions in self-reported pain associated with nature interventions. The estimated mean effect size (SMD 0.535, *P* = 0.000000029) and its 95% CI are shown as a diamond based on *k* = 96 effects from *n* = 62 studies. The dotted line indicates the prediction interval for the estimated mean effect size (−0.78 to 1.85). The estimated mean effect is based on a three-level intercept-only meta-analysis with RVE (two sided). The annotations D1–D5 correspond to the domains of the Cochrane Risk of Bias assessment. O, overall bias. Note that the risk of bias was assessed at the individual level of the studies. The risk of bias for each domain is color coded: green represents low concern, yellow represents some concern and red represents high concern. Source data

We conducted a three-level intercept-only meta-analysis with robust variance estimation (RVE), specifying random effects for individual effect sizes nested within studies to estimate the overall effect of nature exposure on self-reported pain. We found a significant estimated standardized mean effect size of standard mean difference (SMD) 0.535, 95% confidence interval (CI) 0.37 to 0.70, *t*(59.4) = 6.37, *P* < 0.001. On average, nature exposure was associated with lower self-reported pain relative to comparators, corresponding to a reduction of 1.08 points on commonly used 0–10 pain scales. Figure 2 displays the forest plot for all 96 individual effect estimates (Supplementary Fig. 1 shows a plot using within-study aggregated estimates). We found substantial heterogeneity across and within studies. The test for heterogeneity was significant (*Q*(95) = 1,053.74, *P* < 0.001) with a broad prediction interval (95%) for the SMD (−0.78 to 1.85). The *I*^2^ index indicated high heterogeneity (95.9%), with 28.5% attributed to within-study and 67.4% to between-study variance. Comparing the original model with models that constrained level 2 (within-study) or level 3 (between-study), variances revealed significant within-study, ${\chi }^{2}$(2) = 13.43, *P* < 0.001 and between-study heterogeneity, ${\chi }^{2}$(2) = 323.30, *P* < 0.001.

To assess the robustness of the overall effect and explore sources of heterogeneity, we conducted sensitivity analyses by excluding outliers, influential cases or studies measuring spontaneous chronic pain. All adjusted models yielded significant mean effect sizes, although with reduced magnitude (Supplementary Results). When excluding outliers—conservatively defined as effects whose 95% CIs did not overlap with the pooled effect CI—the SMD decreased to 0.485 (95% CI 0.40 to 0.56), with a substantially narrower prediction interval of 0.07 to 0.90. Similarly, removing influential cases identified via Cook’s distance and DFBETAs reduced the SMD to 0.447 (95% CI 0.34 to 0.55) and narrowed the prediction interval to −0.27 to 1.17. These results suggest that a notable portion of the heterogeneity stemmed from influential and outlier cases.

**Fig. 3:**
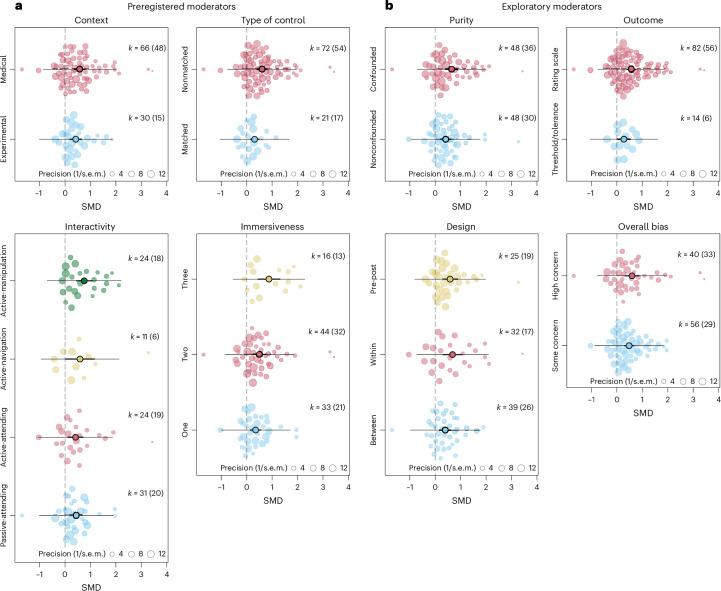
Orchard plots of effect sizes grouped by categorical moderators. **a**, Orchard plots depicting effect sizes grouped by preregistered categorical moderators. **b**, Orchard plots depicting effect sizes grouped by exploratory categorical moderators. Each dot represents an individual effect size estimate, with its size indicating the precision (inverse standard error) of the estimate. Estimates are based on three-level intercept-only meta-analysis with RVE using the respective variables as moderators (two sided). *k* denotes the number of individual effect sizes per moderator category, with the number of contributing studies (*n*) shown in parentheses. Black dots indicate estimated mean effect sizes, with thick error bars representing 95% CIs and thin error bars representing prediction intervals. Estimated mean effect sizes and CIs are based on the following sample sizes: for **a**: medical (*k* = 66, *n* = 48), experimental (*k* = 30, *n* = 15), nonmatched (*k* = 72, *n* = 54), matched (*k* = 21, *n* = 17), active-manipulation (*k* = 24, *n* = 18), active-navigation (k = 11, *n* = 6), active-attending (*k* = 24, *n* = 19), passive-attending (*k* = 31, *n* = 20), three (*k* = 16, *n* = 13), two (*k* = 44, *n* = 32), one (*k* = 33, *n* = 21); for **b**: confounded (*k* = 48, *n* = 36), nonconfounded (*k* = 48, *n* = 30), rating scale (*k* = 82, *n* = 56), threshold/tolerance (*k* = 14, *n* = 6), pre–post (*k* = 25, *n* = 19), within (*k* = 32, *n* = 17), between (*k* = 39, *n* = 26), high concern (*k* = 40, *n* = 33), some concern (*k* = 56, *n* = 29). Source data

To assess potential sources of heterogeneity, we conducted ten unimoderator meta-regression models (four preregistered and six exploratory) each testing the moderating effect of a single variable. Table 2 presents the overall test of moderation for each variable, along with category-specific estimates for categorical moderators and regression coefficients for continuous moderators. Orchard plots illustrating differences in levels of categorical moderators are shown in Fig. 3.

**Table 2:** Moderator analyses separated by preregistered and exploratory moderator variables

Moderator	Test of overall moderator effect		
		Categorical moderator	SMD (95% CI)
Preregistered			
Context	*F*(1, 18.9) = 1.07, *P* = 0.312	Experimental Medical	0.42 (0.17 to 0.67) 0.57 (0.35 to 0.80)
Interactivity	*F*(3, 10.9) = 0.96, *P* = 0.445	Passive-attending Active-attending Active-navigation Active-manipulation	0.44 (0.18 to 0.69) 0.41 (0.11 to 0.72) 0.59 (0.01 to 1.17) 0.75 (0.44 to 1.07)
Immersiveness	*F*(2, 22.4) = 2.52, *P* = 0.102	One modality Two modalities Three modalities	0.35 (0.13 to 0.57) 0.51 (0.25 to 0.76) 0.87 (0.43 to 1.32)
Type of control	*F*(1, 16.2) = 9.01, *P* = 0.008	Matched Nonmatched	0.31 (0.13 to 0.49) 0.61 (0.41 to 0.81)
Exploratory			
Design	*F*(2, 39.2) = 0.99, *P* = 0.378	Between Within Pre–post control	0.39 (0.16 to 0.64) 0.59 (0.28 to 0.89) 0.68 (0.29 to 1.07)
Type of outcome	*F*(1, 6.4) = 3.15, *P* = 0.123	Threshold/tolerance Scale	0.28 (0.01 to 0.55) 0.57 (0.38 to 0.76)
Purity	*F*(1, 26.9) = 2.58, *P* = 0.120	Nonconfounded Confounded	0.41 (0.21 to 0.62) 0.64 (0.40 to 0.89)
Overall bias level	*F*(1, 58.7) = 0.46, *P* = 0.498	Some concern High concern	0.48 (0.27 to 0.68) 0.59 (0.32 to 0.87)
		Continuous moderator	*β* (95% CI)
Publication year	*F*(1, 12.9) = 0.51, *P*= 0.487	Year (centered)	−0.05 (−0.21 to 0.11)
Standard error	*F*(1, 19.7) = 24.5, *P* < 0.001	s.e.m. (centered)	0.47 (0.27 to 0.67)

Owing to missing or ambiguous data we excluded effect sizes for the following variables: interactivity (six studies excluded), immersiveness (three studies excluded) and type of control (three studies excluded). Furthermore, we excluded effect sizes for the studies with immersiveness coded as ‘4’ (three studies), reflecting interventions conducted in real outdoor settings where the number of sensory modalities involved was unclear. The estimated mean effects are based on a three-level intercept-only meta-analysis with RVE using the respective variable as a moderator (two sided). *P* values across moderators were not adjusted for multiple comparisons. The exact *P* value for the moderator ‘standard error’ is *P* = 0.00000000082.

We found no significant moderation effects for the following variables: study context (medical versus experimental; preregistered), interactivity (passive-attending versus active-attending versus active-navigation versus active-manipulation, preregistered), study design (between-participant, within-participant or pre–post control group designs, not preregistered), type of outcome (rating scales versus tolerance or threshold measures, not preregistered) or purity (confounded versus nonconfounded, not preregistered). However, we observed two moderators that had an impact on the overall effect size. First, type of control (preregistered) significantly moderated the effect (*F*(1, 16.2) = 9.01, *P* = 0.008; *Q*(91) = 987.04, *P* < 0.001). Nature interventions were roughly half as effective when compared to matched comparators (*k* = 21, SMD 0.31, 95% CI 0.13 to 0.49, *t*(18.97) = 3.60, *P* = 0.002) as when compared to nonmatched comparators (for example TAU, *k* = 72, SMD 0.61, 95% CI 0.41 to 0.81, *t*(16.19) = 3.00, *P* < 0.001; Fig. 3a and Table 2). Second, while the overall moderator test for immersiveness (number of sensory modalities; preregistered) was not statistically significant (*F*(1, 22.4) = 2.52, *P* = 0.102; *Q*(90) = 956.48, *P* < 0.001), studies targeting three modalities showed a significantly larger effect compared to studies targeting one modality (*β* = 0.52, *P* = 0.04, 95% CI 0.03 to 1.02) with an estimated SMD of 0.87 versus 0.35, respectively (Fig. 3a and Table 2).

**Fig. 4:**
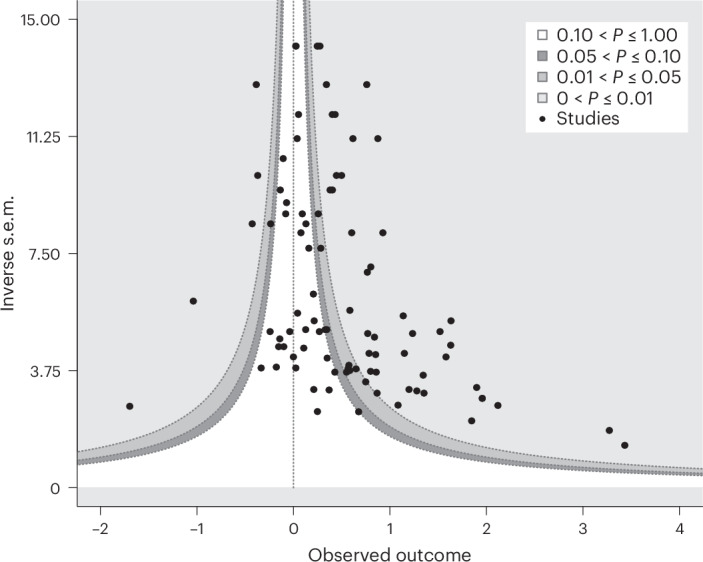
Contour-enhanced funnel plot displaying individual effect sizes (SMD) plotted against their inverse standard errors. Each dot represents a single effect size from the studies included in the meta-analysis. The shaded regions indicate conventional thresholds of statistical significance (*P* < 0.05, *P* < 0.01, *P* < 0.001; two sided), which serve as visual guides to assess potential small-study effects or publication bias. The vertical line at a SMD of 0 indicates no effect of nature interventions on self-reported pain. No formal statistical test for funnel plot asymmetry was conducted, as this plot is intended for qualitative assessment only (for further details, see Supplementary Methods). Source data

In addition, we conducted moderation analyses, including measures of bias, and found that out of three bias-related variables, two were nonsignificant moderators. First, there was no difference between studies with ‘some’ versus ‘high’ risk of bias (not preregistered) and second, we did not find an effect of publication year (not preregistered). When including standard error of the effect size as a moderator (a common proxy for small-study effects and funnel plot asymmetry) we found a significant positive association with effect size (*β* = 0.473, s.e.m. 0.095, *t*(19.7) = 4.95, *P* < 0.001), suggesting that studies with lower precision tended to report larger effects. Visual inspection of the contour-enhanced funnel plot^42^ showed asymmetry favoring studies that reported reductions in pain from nature exposure (Fig. 4). However, the presence of numerous nonsignificant effects indicated limited evidence for publication bias based solely on statistical significance.

## Discussion

This preregistered systematic review and meta-analysis assessed whether nature exposure reduces self-reported acute pain or spontaneously occurring chronic pain. We found evidence for a small-to-moderate analgesic effect across published studies conducted in diverse settings, countries and employing various experimental and clinical procedures, suggesting that nature exposure can indeed reduce pain. However, substantial heterogeneity in study design and outcomes led to considerable variation in effect sizes, indicating that effectiveness depends on contextual and methodological factors. Examining this heterogeneity using moderator analyses, we did not find significant effects for study context, stimulus interactivity, study design, outcome type, purity or overall risk of bias. However, effects differed by comparator type (matched versus nonmatched) and number of sensory modalities targeted. Evidence that smaller studies tended to report larger effects, combined with widespread risk of bias, underscores the need for caution when interpreting the specificity and magnitude of these findings.

The observed small-to-moderate estimated mean effect (SMD 0.535) corresponds to an approximate reduction of 1.08 points on a 0–10 pain rating scale. This magnitude is comparable to other nonpharmacological interventions, including behavioral or exercise therapy^43^, music interventions^44,45^ and some distraction techniques for children^46^. It is also similar to analgesic responses of three to four standard alcoholic drinks^47^ or cannabinoid administration^48^. However, it is considerably smaller than the short-term pain reductions typically observed with opioid or nonopioid treatments, which can decrease self-reported pain by three to four scale points^49,50^. Although statistically significant, the effect falls short of the two-point threshold for “moderately important improvements” according to IMMPACT guidelines, but qualifies as a ‘minimally important difference’^51^, supporting its clinical meaningfulness. Importantly, given the low-cost, minimal risk and potential scalability of nature-based interventions, even this minimally important difference holds considerable potential for meaningful population-level benefits. Our findings thus suggest that nature exposure serves best as an adjunct or complementary approach to pharmacological strategies since it may reduce the required dose of analgesics or enable the substitution of stronger medications with weaker ones^16,52,53^ (but see refs. ^31,54^). This can help mitigate dose escalations, a process of gradually increasing dosages to maintain pain relief, which is often ineffective in alleviating subjective pain in the medium-to-long term^55^ and is associated with increased adverse outcomes. Consequently, it could potentially help to disrupt cycles of medication overuse or abuse.

In addition, nature exposure could alleviate the broader burden of pain by targeting mental health symptoms that commonly co-occur with pain-related conditions. Pain and mental health are closely interlinked, with both acute and chronic pain associated with various mental health challenges, including depression, catastrophizing, substance abuse, fear or anxiety^7,56^. For example, anxiety not only exacerbates acute pain^57,58^ but is also a strong predictor of chronic postsurgical pain^59^. Conversely, persistent and intense pain elevates the risk for chronic anxiety disorders^60^. Similar bidirectional associations have been suggested for depression and catastrophizing^60^. Nature exposure has been linked to a reduction in mental health symptoms, including anxiety^61^, depression^62^ or rumination^63^. In our meta-analysis, half of the studies assessed state anxiety as a secondary outcome. Of these, 61.3% (*n* = 19) reported reductions in anxiety following nature exposure^27,28,33,40,64–75^, even when pain levels remained unchanged^76–78^. By contrast, 32.3% (*n* = 10) found no effect and 6.4% (*n* = 2) reported adverse effects^31,32,37,52,79–84^. Moreover, recent evidence suggests that access to urban greenspace may buffer the association between pain catastrophizing and intensity in patients^85^. Together, these findings indicate that nature exposure may provide broader biopsychosocial benefits, addressing interrelated mental and physical dimensions of pain.

Given the substantial heterogeneity across studies, along with the risk of bias assessment discussed below, our findings warrant cautious interpretation and point to low certainty of the evidence (Supplementary Results), highlighting the need for more rigorous and homogeneous research. Notably, sensitivity analyses excluding outliers and influential cases yielded markedly narrower prediction intervals, indicating that some heterogeneity was driven by atypical effects. This is especially relevant given that estimates derived from several studies exceeded large (SMD >1) to very large (SMD >2) effect sizes, raising concerns about potential overestimation. However, excluding most of these studies based on outlier or influential diagnostics resulted in an estimated mean effect comparable to that of the full set of studies, thus supporting the robustness of the overall finding. To further investigate possible sources of heterogeneity, we conducted unimoderator analyses. Two preregistered moderators emerged as potentially relevant: the type of comparator (matched versus nonmatched) and the immersiveness (number of senses engaged).

Studies comparing nature interventions to nonmatched comparators (for example, TAU or no stimulation) revealed nearly double the effect size of those using relatively matched comparators (for example, non-natural urban environments). Importantly, while the estimated mean effects from both study categories differed significantly from zero, this finding suggests that more rigorous designs are needed to more accurately assess nature’s potential impact. Furthermore, interventions targeting three modalities showed over twice the effect size compared to those targeting just one, suggesting that richer, multisensory approaches may enhance analgesic outcomes. Rather than relying on, for instance, two-dimensional (2D) displays of images, interventions should incorporate sounds, scents or interactive elements. Only three studies exposed participants to ‘real’ nature, which may offer the greatest benefits by inherently engaging multiple senses. Where ‘real’ nature is unfeasible (for example, medical environments), VR presents a promising alternative^86^. Studies comparing 2D and VR presentations of the same stimuli generally found stronger effects^28,31,72,87^ with VR (but see refs. ^29,88^) and among the ten largest effects, many involved VR delivery in clinical settings. However, VR has limitations, including motion sickness, cost and limited availability in socioeconomically disadvantaged populations. Notwithstanding these challenges, increasing the immersiveness of nature experiences may be a feasible way to amplify its analgesic potential.

Other potential moderators, such as interactivity, type of outcome, the presence of confounding elements and study design or context, did not demonstrate significant effects. First, contrary to our preregistered hypothesis, stimulus interactivity showed no moderating effect. However, its strong association with immersiveness (*ω* = 0.92) complicates the interpretation, as it probably reflects substantial shared variance between the two variables (for a complete overview of pairwise associations among moderators, see Supplementary Results). Moreover, only one study directly investigated interactivity^15^, highlighting the need for focused research on this factor. Second, studies employing tolerance or threshold measures tended to report smaller effects than those using pain scales, but this comparison did not reach significance. Third, interventions confounded by additional elements (for example, music, meditation and hypnosis) showed larger effects than nature alone, implying these elements may amplify analgesic outcomes. Yet the lack of significant differences suggests that nature exposure by itself was effective and not impacted severely by these elements. Fourth, no significant variation in effect sizes emerged across different study designs, indicating comparable treatment effect estimation^89^. Last, analyses showed no significant difference between experimental or medical settings, indicating that nature exposure was similarly effective across experimental methods and medical settings. However, the available evidence limited deeper exploration of how specific subtypes of medical and experimental pain procedures might be differentially impacted. For example, low analgesic effects were seen in studies involving medical procedures eliciting minimal self-reported pain^30,65,76^ (for example, blood draws or chemotherapy infusion), probably owing to bottom effects. Further research should clarify which pain conditions are most responsive to nature to better explain interstudy variability.

Beyond concerns about generalizability, the studies can be characterized as carrying considerable risk of bias. Specifically, using the Cochrane risk of bias tool^41^ indicated that all included studies exhibited some concern or high risk of bias. However, a moderator analysis found no significant differences in effect sizes based on overall bias classification, suggesting that methodological shortcomings did not substantially impact the overall effect. Nevertheless, the prevalence of bias calls for caution in interpreting the results. Notably, over half of the included studies raised concerns about randomization and failed to report on allocation concealment. Furthermore, the absence of preregistration in most studies limited transparency around outcome selection and selective reporting. While this is unsurprising for research conducted in earlier decades when (pre)registration was less common, it highlights an important methodological shortcoming. Moving forward, a greater adherence to open science practices, including preregistration, transparent reporting and data sharing, is necessary to strengthen the evidence base.

A key consideration was risk of bias related to outcome measurement, with all but two studies rated as potentially biased. According to the Cochrane risk of bias tool, studies where knowledge of the intervention by the participants could influence self-reported outcomes were categorized as having some or high concern, since self-reported pain is inherently vulnerable to expectancy effects, placebo responses and demand characteristics. These challenges are not unique to nature exposure research. For instance, up to 50% of self-reported analgesic effects in pharmacological agents have been linked to expectancy mechanisms^90^. Given the experiential and inherently subjective nature of pain, self-reports remain not only valuable but are key to capturing the essence of pain as a subjective experience. However, increased effort should be invested to complement them with more objective and mechanistic measures. For instance, psychophysiology or neuroimaging methods offer useful alternatives for measuring pain. Notably, several studies have collected additional data on pulse and heart rate or their variability^25,28,30,31,33,72,77,81,83,84,91–95^, blood pressure^30,54,77,81,83,84,92,93,96–98^, respiratory rate^72,84,91,94–96^ and skin conductance^25,28,33^. Only a few studies have utilized neuroimaging methods such as electroencephalography^87,99,100^ or functional magnetic resonance imaging (fMRI). Leveraging neuroscience can complement self-reports and uncover the underlying mechanisms driving nature’s analgesic effects. For example, a recent preregistered fMRI study from our group (published after the study inclusion cutoff of this review), found that exposure to nature, compared to well-matched urban or indoor environments, reduced both self-reported pain and brain responses linked to lower-level nociceptive processing^101^. These results indicated that nature’s analgesic effects may involve neural pathways distinct from those implicated in expectancy or placebo responses. Similarly, another fMRI study showed that virtual nature lessened secondary hyperalgesia and was associated with altered insulo-thalamic effective connectivity^102^. Future well-controlled, mechanistic studies incorporating primary and secondary pain outcomes, less susceptible to bias, are critical to guide the development of optimized interventions with meaningful clinical impact and clarify the specific neural mechanisms involved.

Nature’s analgesic effects may stem from various psychological and physiological mechanisms, which remain largely unexplored. One likely mechanism is attentional modulation. Attention restoration theory^103^ suggests that certain natural elements are highly engaging and may redirect attentional resources away from pain more effectively than other stimuli. Supporting this, our recent neuroimaging work linked nature exposure to reduced activity in sensory-discriminative brain regions involved in nociceptive signaling, paralleling findings from mindfulness-based interventions, which similarly modulate attention-related neural circuits^101^. Stress recovery theory^104^ provides an alternative perspective, proposing that specific aspects of nature induce positive affective changes and reduce stress, both known to modulate pain^105,106^. Both frameworks suggest that nature is not intrinsically analgesic but facilitates pain relief through changes in cognitive and affective processes that can also be targeted by other interventions, such as mindfulness-based stress reduction^107,108^ or cognitive behavioral therapy^109^. Nature may differ from these approaches in its low cognitive demand and accessibility, evoking regulatory effects with minimal effort or training. Further studies should directly compare nature exposure with established psychological treatments and integrate environmental psychology theories with pain research^110^ to clarify its mechanisms of action.

Future research should also identify which elements of nature are most effective for pain relief and which individual differences are important. It remains uncertain whether features known to improve mood and restoration, such as biotic and abiotic sounds^111,112^, the presence of water^113^, environmental complexity^114^ and perceived possibilities for prospect or refuge^115^, also enhance nature’s analgesic effect. Aside from one study showing no pain differences across prospect–refuge combinations^116^, their specific role in pain modulation remains underexplored. Benefits may also vary across subgroups. For instance, individuals who feel psychologically closer to nature may experience greater pain relief^117^. It also remains unclear whether these effects extend to children and adolescents as these groups were excluded from this review owing to comparability issues with self-reported pain measures^118^. In addition, identifying which pain types respond best to nature and which environment–pain combinations are particularly effective needs to be established. For example, ‘cold’ environments may benefit burn-injured patients more than ‘warm’ ones^119^. Last, nearly all the summarized evidence focused on acute pain, which offers greater experimental control but limits generalizability to chronic pain^120^, highlighting the need for future studies on nature’s impact in chronic pain populations.

Beyond the limited generalizability to chronic pain, the limitations discussed earlier critically shape the interpretation of our findings. The certainty of the findings is constrained by substantial heterogeneity across studies and their generally modest methodological quality. While the wide prediction interval reflects considerable inconsistency, sensitivity analyses showed that excluding outliers and extreme cases reduced this inconsistency without altering the estimated mean effect. The moderate-to-high risk-of-bias assessments further underscores the need for more rigorous primary research in this field. Notably, the risk-of-bias assessment was largely driven by the reliance on subjective pain ratings as the primary outcome, which are inherently vulnerable to various forms of bias. Our focus on subjective outcomes was justified by the following considerations. Pain is an inherently personal experience^121^, and thus requires some sort of subjective report to capture how it is experienced by a person. This is why self-reported ratings are the widely accepted standard in pain research and clinical practice^51^. Their frequent use across studies also enabled the synthesis of a broad evidence base. However, future research should complement subjective ratings with additional measurements and experimental manipulations to address bias and provide deeper insights into the mechanisms underlying these effects. Last, because this study relied solely on secondary, anonymized data from previously published studies, detailed information on sex or gender was not available for all studies. We have summarized the number of female and male participants in the methods section. However, it was not feasible to conduct separate analyses based on sex or gender.

## Conclusions

Nature exposure is associated with a small-to-moderate reduction in self-reported acute or spontaneous chronic pain across diverse studies. However, substantial heterogeneity and potential bias warrant cautious interpretation and call for more rigorous research to clarify the effect’s generalizability, specificity and underlying mechanisms. Interventions appeared most beneficial with multisensory stimuli and when contrasted against nonmatched comparators, suggesting that context and methodological choices shape outcomes. Although evidence for the analgesic effects of nature is growing, further studies are needed. Their emphasis should expand beyond acute pain, which was the primary focus here, to chronic and recurrent pain, and examine how individual-level factors and characteristics of nature influence its impact. We particularly advocate for rigorous studies adhering to open science practices and integrating both subjective and objective measures of pain. Such research will help uncover underlying mechanisms and optimize nature-based approaches as complementary pain-management strategies. Given the high global burden of pain, showing that such complementary, accessible and scalable interventions may reduce pain represents an important step toward expanding nonpharmacological treatment options and improving patient outcomes.

## Methods

This systematic review and meta-analysis followed the guidelines of the Cochrane Handbook for Systematic Reviews of Interventions^122^ and the PRISMA 2020 statement^123^. The completed PRISMA checklist is included at the end of the Supplementary Information. We preregistered the systematic review and meta-analysis on PROSPERO (reference ID CRD42023478942) and report both preregistered and exploratory analyses.

### Eligibility criteria

We included studies involving adult participants from healthy and clinical populations undergoing experimental or medical procedures typically experienced as painful. Studies had to feature at least one intervention that stimulated one or more sensory modalities (for example, visual, auditory, tactile) primarily using natural stimuli—such as videos of natural scenes, natural soundscapes or tactile interaction with natural materials—and include some form of comparator (matched or nonmatched; see below). Although this broad inclusion criterion introduced variability in the specific natural stimuli and delivery formats, the unifying feature across all interventions was that nature-based sensory input was the central component of the intervention. Self-reported pain was required as an outcome, assessed using either scales (VAS, NRS or GRS) or measures of pain threshold or tolerance. When multiple pain dimensions were assessed, we prioritized self-reported pain intensity as it was the most consistently reported measure across studies, allowing for the inclusion of the broadest possible dataset. We selected those closest to the painful stimulation for studies with multiple ratings at different time points. We included studies with between-participant, within-participant or pre–post control group designs. Eligible studies had to be published in English, peer-reviewed journals and present original research (excluding opinion pieces, reviews and so on). Studies were deemed ineligible if they employed inadequate interventions, comparators, and outcomes or provided insufficient data (Supplementary Methods and Supplementary Table 1). Furthermore, we excluded gray literature (for example, dissertations, conference abstracts and so on).

### Study search, selection and coding

We searched the extent of the literature up until 31 January 2024 (no restrictions on earlier publication date) using four different databases: PsycINFO, PubMed, Web of Science and SCOPUS. We combined terms related to nature interventions, pain outcomes and exclusion criteria (Supplementary Methods). The same terms were used across all four databases, with adjustments made for database-specific search rules. In addition, we performed reference tracing through forward and backward tracking. Two reviewers (M.O.S. and J.P.N.) independently reviewed, coded (for example, study characteristics, moderators, risk of bias and certainty) and extracted data from all studies. Initially, we assessed titles and abstracts against our inclusion criteria, excluding those that did not meet them. We then reviewed the full text of the remaining articles. Discrepancies and ambiguities were resolved through direct discussion between the reviewers. The final sample included 62 studies encompassing a total sample size of *N* = 4,439 participants. Data on sex/gender was reported in 58 out of 62 studies from which 1,989 (44.98%) were male and 2,433 were female (55.02%). Mean age was reported in 52 out of 62 studies and ranged from 20.33 to 70.94 years, with a weighted arithmetic mean of 44.91 years. For a PRISMA flow chart, see Fig. 1.

To investigate heterogeneity in intervention characteristics and study designs, we coded the studies for several factors and used these in moderator analyses. This allowed us to explore whether different forms of nature exposure or study features moderated the observed effects. To this end, we coded the studies for eight categorical factors (Supplementary Methods). First, we differentiated between medical (that is, clinical) and experimental studies. Second, we coded the level of interactivity of the nature interventions using four levels: passive-attending, active-attending, active-navigation and active-manipulation. Third, we coded the level of immersiveness of the nature interventions, representing the number of sensory modalities engaged. Fourth, we coded comparators as either matched or nonmatched. Matched comparators were active controls, such as a non-nature stimulus presented through the same or a similar medium as the nature stimulus or any other intervention participants might reasonably construe as an active intervention (for example, squeezing a stress ball). Nonmatched comparators included control conditions that did not offer additional stimulation (for example, TAU or turned-off devices such as blank screens or headphones without audio). Fifth, we assessed the study design type and differentiated between-participant, within-participant and pre–post control group designs. Sixth, we coded nature interventions as either pure or non-pure. Non-pure interventions included additional and potentially confounding elements such as relaxing music or autohypnosis. Pure interventions consisted solely of nature elements. Seventh, we coded the type of outcome to distinguish between studies measuring pain with scales (NRS, GRS and VAS) and those measuring pain through threshold and tolerance assessments. Eighth, we differentiated between studies with some or high risk of bias based on the revised Cochrane risk of bias tool. For an exhaustive list of all studies, including additional extracted details such as moderator coding or the location of the original data within each article, see Supplementary Table 2.

### Data synthesis and analysis

We extracted means and s.d. from the text or raw data whenever possible. We contacted the study’s first and last authors if no values were available. We converted medians, quartiles, interquartile ranges or s.e.m. to means or s.d. using established methods^124–126^. If none of this information was provided but the article included figures, we manually extracted data using WebPlotDigitizer^127^. We conducted all analyses in R (v4.2.1; R Core Team 2022) using the packages metafor^128^ and ClubSandwich^129^. We used SMD as effect sizes (for details, see Supplementary Methods). To ensure the comparability of SMDs across different study designs^89,130^, all SMDs were based on raw score metrics calculated using the escalc() function from the metafor package. We performed a three-level intercept-only meta-analysis and used RVE to estimate the s.e.m. and CIs of the fixed effects^131^. We chose this approach to address dependencies in effect sizes and sampling errors, as multiple effect sizes within our dataset originated from the same study or included overlapping samples. Compared to traditional methods, multilevel meta-analysis and RVE are more effective for handling these dependencies^132,133^. Random effects were specified for individual effect sizes nested within studies, and we used the vcalc() function of the metafor package to approximate the variance–covariance matrix of the sampling errors of dependent effect sizes. After running the main model, we performed sensitivity analyses by excluding outliers, influential cases and studies assessing spontaneous chronic pain^134^.

We calculated Cochran’s *Q* test and the *I*^2^ statistics to assess heterogeneity, assessed the within and between-study variance for significance and interpreted the *I*^2^ value according to recommended guidelines^132,135,136^. Figures 2–4 were created using the packages metafor^128^ and orchaRd^137^. Details on specifications of the main model, sensitivity analyses, heterogeneity and funnel plot asymmetry are reported in the Supplementary Information. All statistical tests were two sided.

### Moderator analyses

We conducted moderation analyses using ten variables: (1) context, (2) interactivity (3) immersiveness, (4) type of control, (5) study design, (6) type of outcome, (7) purity, and (8) overall bias, (9) publication year and (10) the standard error of effect sizes. This last moderator analysis was used as a test for funnel plot asymmetry and can also be regarded as an Egger’s test for multilevel meta-analysis, as suggested by Rodgers and Pustejovsky^138^. The first four variables were preregistered. First, we examined the influence of study context by comparing medical versus experimental settings. Second and third, we assessed the impact of interactivity and immersiveness of the nature intervention. Fourth, we compared outcomes based on the type of control condition, distinguishing between matched and nonmatched comparators. The remaining six variables were not preregistered. They were added after collecting and reviewing all eligible articles, as we identified their relevance only after familiarizing ourselves with the existing literature. Accordingly, we investigated different study design types by contrasting between-participant, within-participant and pre–post control group designs. Furthermore, we differentiated between studies using pain rating scales and those employing pain threshold and tolerance measures. Pain rating scales, commonly used to assess self-reports of pain, typically require participants to select a value between ‘no pain’ to ‘worst pain possible’. By contrast, pain threshold and tolerance measures assess the time it takes for participants to identify a stimulus as painful (threshold) or too intense to bear (tolerance). While both methods operationalize subjective pain experiences, they differ in their approaches to measurement and capture slightly different aspects of the pain experience. We therefore evaluated their relevance in this review. In addition, we contrasted pure nature interventions with those potentially confounded by additional elements. Furthermore, we investigated the impact of overall study bias by comparing studies with some or high risk of bias. Last, we examined the effect of publication year (centered) and precision (standard error; centered) of the effect size. For a detailed description of moderator coding, see the Supplementary Methods.

### Risk of bias

Risk of bias was evaluated for each included study using the revised Cochrane risk of bias tool^41^ covering five domains: (D1) randomization process, (D2) deviations from intended interventions, (D3) missing outcome data, (D4) measurement of the outcome and (D5) selection of reported results. We also assessed the overall risk of bias according to the toolbox guidelines, where studies with ‘some’ or ‘high’ concern in at least one domain were given an overall assessment of ‘some’ or ‘high’ concern, respectively.

### Reporting summary

Further information on research design is available in the Nature Portfolio Reporting Summary linked to this article.

## Supplementary information

Supplementary Information Supplementary Methods, Results, Tables 1–4, Figs. 1 and 2, References and PRISMA 2020 checklist.

Reporting Summary

Supplementary Code e2 Source code for recreating Fig. 2.

Supplementary Code e3 Source code for recreating Fig. 3.

Supplementary Code e4 Source code for recreating Fig. 4.

Supplementary Code 1 Source code for recreating Supplementary Fig. 1.

Supplementary Code 2 Source code for recreating Supplementary Fig. 2.

## Source data

Source Data Figs. 2–4 Source data for Figs. 2–4, and Supplementary Figs. 1–2.

## Data Availability

The extracted data, including means, standard deviations, sample sizes, study and effect IDs, and moderator coding, are available via OSF at https://osf.io/hmf4r/. Source data are provided with this paper.

## Code availability

The code used for the meta-analysis, including all supplementary analyses, is available via OSF at https://osf.io/hmf4r/. In addition, the code for reproducing all figures is provided alongside the Source data.
