# Supplementary material for: Nature exposure reduces self-reported pain: a systematic review and meta-analysis
Source: Nat Ment Health. 2026 Jan 6;4(1):165–80. doi: 10.1038/s44220-025-00569-2 (PMC12789040; doi:10.1038/s44220-025-00569-2)
Supplement: Supplementary file 1 — Supplementary Methods, Results, Tables 1–4, Figs. 1 and 2, References and PRISMA 2020 checklist. [file 44220_2025_569_MOESM1_ESM.pdf]

nature mental health

Supplementary information

https://doi.org/10.1038/s44220-025-00569-2

Nature exposure reduces self-reported pain: a systematic review and meta-analysis

In the format provided by the authors and unedited

Table of contents

SUPPLEMENTARY METHODS 2
SUPPLEMENTARY RESULTS 5
SUPPLEMENTARY TABLE 1 8
SUPPLEMENTARY TABLE 2. 9
SUPPLEMENTARY TABLE 3 19
SUPPLEMENTARY TABLE 4 20
SUPPLEMENTARY FIGURE 1 21
SUPPLEMENTARY FIGURE 2 22
SUPPLEMENTARY REFERENCES 23
PRISMA 2020 CHECKLIST 30

Supplementary Methods

Study search. For our study search, we utilized four databases (PsycINFO, PubMed, Web of Science, and SCOPUS) and combined multiple search terms tailored to the specific search rules of each database. For instance, in Web of Science, we employed the following Boolean search query: ALL=(("Natur* Scen*" OR "Environment* Scen*" OR "Natur* Sound*" OR "Environment* Sound*" OR "Natur* Imag*" OR "Environment* Imag*" OR "Natur* View*" OR "Environment* View*" OR "Scen* View*" OR "Scen* Beauty" OR "Natur* Stimul*" OR "Environment* Stimul*" OR "Biophil*" OR "Natur* Exposure" OR "Natur* Base*" OR "Virtual Natur*" OR "Natur* Contact" OR (("Virtual Reality" OR "VR") AND ("Natur*" OR "Environment*" OR "Distract*"))) AND ("Pain" OR "Pain Tolerance" OR "Pain Threshold" OR "Pain Control" OR "Pain Management") NOT ("Child*" OR "Pediat*" OR "Newborn" OR "Infant") NOT ("Animal*")). Note that we also included the broad keyword “Virtual Reality” (VR) in combination with “Nature”, “Environment” or “Distraction”, as our initial non-systematic review of the literature identified several studies that did not explicitly reference the natural content of the used VR applications. Notably, several studies employed VR scenarios featuring nature content, which appears to be a commonly chosen default among many research groups but is often not explicitly acknowledged. We aimed to include these studies, which utilized stimuli or distractions encompassing natural content while not explicitly referencing nature in their titles or abstracts. Additionally, we restricted the search to peer-reviewed articles in English language.

Study exclusion and inclusion. After identifying records from databases, we removed duplicates and case reports. Based on text review, we tabulated study characteristics and compared them against our predefined PICOs (Population, Intervention, Comparator, Outcome). Supplementary Table 1 lists 23 studies that passed the initial screening (title and abstract) but were excluded after full-text examination. Reasons for exclusion varied and encompassed insufficient control conditions, inadequate data presentation, lack of access to full text, insufficient outcome measures, and inadequate interventions. Despite their exclusion, many of these studies offer valuable insights into the analgesic effects of nature. All remaining studies met the inclusion criteria and were included in the systematic review. All of these reported the primary outcome in a format suitable for quantitative synthesis and were therefore included in the meta-analysis.

Study coding. Studies were coded according to eight criteria. First, we differentiated between *medical* (i.e., clinical) and *experimental* studies. Second, we coded the level of *interactivity* of the nature interventions using four levels: *passive-attending*, *active-attending*, *active-navigation*, and *active-manipulation*. We coded an intervention as *passive-attending* if participants could passively observe it without interaction. We coded an intervention as *active-attending* if participants could actively perceive the environment through 360° views of static images or videos, allowing them to explore the surroundings by moving their heads. We coded an intervention as *active-navigation* if participants could navigate an environment through movement (e.g., by using controllers in VR settings). Lastly, we coded an intervention as *active-manipulation* if participants could interact with a stimulus through direct manipulation, such as touching or moving (and often feeling) it with their hands. Third, we coded the level of *immersiveness* of the nature interventions according to the number of sensory modalities engaged by the stimuli. For example, an intervention involving nature soundscapes was coded as “one”, a nature movie with sound was coded as “two”, and interventions that included visual, auditory, and olfactory elements were coded as

"three". We assigned the highest coding (i.e., four) to studies that exposed participants to "real" nature, as we expected that real outdoor conditions simultaneously engage multiple sensory modalities. Fourth, we coded *comparators* as either relatively *matched* or *non-matched*. A relatively *matched* comparator was an active control intervention, such as a non-nature stimulus presented through the same medium as the nature stimulus or any other comparators participant might reasonably construe as an active intervention (e.g., squeezing a stress ball). *Non-matched* comparators included conditions such as treatment as usual, viewing a black screen or fixation cross, or wearing (deactivated) devices, including turned-off headphones or head-mounted displays (HMD). Fifth, we assessed the study design type and differentiated *between-participant*, *within-participant* and *pre-post control group* designs. Sixth, we coded the *type of outcome* to distinguish between studies measuring pain with scales (numerical rating scales, graphical rating scales, visual analogue scales) and those measuring pain through threshold and tolerance assessments. Seventh, we coded nature interventions as *pure* or *non-pure* based on additional non-natural elements. *Non-pure* interventions included secondary and potentially confounding elements, including calming music, video game aspects (e.g., target shooting), guided meditation, autohypnosis, breathing exercises, or narration. *Pure* interventions consisted solely of nature stimuli (e.g., a VR setting of a beach with the sound of the ocean). Eight, we coded *overall study bias* using the Cochrane risk-of-bias tool, classifying studies as having *some* or *high* risk of bias. Furthermore, we assessed the certainty of evidence using the GRADE framework[1], considering five core domains: risk of bias, inconsistency, indirectness, imprecision, and publication bias. We also considered factors that may increase certainty, including large effects, dose-response gradients, and plausible confounding. Certainty was rated as high, moderate, low, or very low, following the guidance in chapters 11 and 12 of the Cochrane Handbook for Systematic Reviews of Interventions[2]. Two reviewers (MOS and JPS) independently assessed certainty using the GRADEpro GDT software[3], with disagreements resolved through discussion. The full set of GRADE assessments and justifications is reported in the Supplementary Results section.

Data analysis. We estimated all SMDs based on raw score metrics to compare effect sizes across different study designs. For studies reporting medians, quartiles, and quantile (interquartile) ranges (n = 8), we first converted to means and standard deviations using the MLN method, implemented via the mln.mean.sd() function from the estmeansd package[4]. This approach was applied to all studies except one[5], where conversion failed due to a reported median of 0. For this study, we estimated the mean and standard deviation using formulas 14 and 15 from Wan et al. (2014)[6]. Based on the means and standard deviations, we calculated standardized mean differences (SMD) for each study. We estimated all SMDs based on raw score metrics to compare effect sizes across different study designs. We used the following approaches. First, we calculated SMD as Hedge's g for between-participant designs[7].

$$\frac{m1i - m2i}{\sqrt{\frac{(n1i-1)sd1i^2 + (n2i-1)sd2i^2}{n1i + n2i - 2}}}$$

Second, we calculated the standardized mean change using raw score standardization with pooled standard deviations for within-participant designs.[8]

$$\frac{m1i - m2i}{\sqrt{\frac{sd1i^2 + sd2i^2}{2}}}$$

Third, we estimated the standardized mean change using raw score standardization for pre-post control group designs.[9,10]

$$\frac{m1i,\mathrm{E} - m2i,\mathrm{E}}{sd1i,\mathrm{E}} - \frac{m1i,\mathrm{C} - m2i,\mathrm{C}}{sd1i,\mathrm{C}}$$

We specified a correlation of φ = 0.9 between repeated measurements for within-participant and pre-post control group studies. This specification was based on past studies that measured self-reported pain at time intervals similar to most studies included in the meta-analysis[11–15]. We deviated from this approach in two studies. First, one between-participant study directly provided an effect size (odds ratio) instead of the raw means and standard deviations[16]. We converted this effect size to Hedge's g[17]. Second, calculating the standardized mean change using raw score standardization for one study following a pre-post control design[18] resulted in a unrealistically high value (SMD = 5.93). For this study, we re-estimated the SMD as Hedge's g using the post-intervention scores, essentially treating the study as a between-participant design, which resulted in a more plausible, although more conservative, estimate (SMD = 0.58).

After extracting the effect sizes, we conducted a three-level intercept-only meta-analysis[19–21]. We employed robust variance estimation (RVE) to estimate the standard errors and confidence intervals of the fixed effects. Given that several included studies reported multiple effect sizes (e.g., due to multiple intervention groups, comparison groups, or repeated measurements within the same participants, etc.), we expected dependencies in effect sizes and sampling errors within our dataset. Such dependencies can lead to biased estimates, and various methods have been proposed to address them[22,23]. Compared with traditional approaches, multilevel meta-analysis and RVE offer strategies for handling these dependency issues without reducing the number of effect sizes incorporated into the analysis[19,20]. The three-level model is particularly advantageous as it accommodates complex hierarchical data structures, decomposing the variance in effect sizes by simultaneously accounting for sampling (level 1), within-study (level 2), and between-study (level 3) variance[21]. Combining three-level meta-analysis with RVE has the added benefit that knowledge of the exact dependence structure between effect sizes is not required, resulting in robust estimations of standard errors and confidence intervals of fixed effects even when the model is potentially misspecified[23]. We included random effects for study-level and effect-level variability. We used the vcalc() function of the metafor package to approximate the variance-covariance matrix of the sampling errors of dependent effect sizes. We specified a dependency of ρ = 0.5 between pain tolerance and threshold measures and of φ = 0.9 between consecutive measurements of self-reported pain. These dependency values were selected based on prior literature investigating the relationship (ρ) of pain threshold and tolerance measures[24–26] and the correlation of repeated self-reported pain measurements (φ) using the rating scales stated above. After running the main model, we performed sensitivity analyses by excluding outliers, influential cases, and two studies assessing spontaneous chronic pain[27]. Importantly, outlier and influential case identification were based on the multilevel model before conducting RVE. After excluding potentially problematic cases, all analyses were rerun using RVE.

Regarding heterogeneity, we assessed the significance of within- and between-study variance by following an approach suggested by Assink and Wibbelink[19]. We performed two separate log-likelihood ratio tests (LRT), comparing the original model with freely estimated level 2 (within) and level 3 (between) variances to two constrained models. Constraining the level 2 (within) or 3 (between) variance and comparing these models to the original model revealed significant results with $\chi^2(2) = 13.43$, $p < .001$ for within-, and $\chi^2(2) = 323.30$, $p < .001$ for between-study heterogeneity, respectively.

To test for funnel plot asymmetry, we followed an approach suggested by Rodgers and Pustejovsky[28]. We reran our multilevel model but included the standard error of the effect sizes as an additional moderator in the model. Egger's test was then performed using the coef_test() function of the package clubSandwich[29]. A significant effect of the moderator variable indicates a relationship between the effect sizes and their respective standard errors, which suggests asymmetry in the funnel plot. Note that funnel plot asymmetry can be due to various factors, including selective reporting or heterogeneity between studies (e.g., due to study design differences).

Supplementary Results

Sensitivity Analyses. We ran several sensitivity analyses to investigate how much outliers, influential cases, or studies assessing spontaneous chronic pain impacted the main model. We identified outliers based on our multilevel model before applying RVE using different strategies suggested in the literature[27,30]. Additionally, we investigated the impact of excluding two studies that assessed spontaneous chronic pain. After excluding the identified cases, we reran the respective multilevel model and applied RVE. Supplementary Table 3 gives an overview of the resulting SMD, along with its 95% confidence interval (CI) and prediction interval (PI), and the number of effect sizes (k) removed after excluding outliers and influential cases. Supplementary Figure 2 compares the distribution of effect sizes from the model including all studies with those from models based on subsets of studies, excluding outliers (a-b), influential cases (c), or studies measuring spontaneously occurring chronic pain (d).

First, we used a very stringent strategy and identified all effect sizes as outliers whose confidence intervals (CI) did not overlap with the CI of the pooled effect. This resulted in the exclusion of 42 effect sizes encompassed in 23 studies. Calculating the multilevel model across the remaining 54 effect sizes from 39 studies resulted in a significant effect of nature on self-reported pain with an estimated mean effect size of SMD = 0.484 (SE = 0.039; 95% CI[0.40, 0.57]), $t(30.9) = 12.17$, $p < .001$ and a prediction interval of [0.069, 0.900]. Second, we used the rstudent() function of the metafor[31] package to obtain (externally) studentized residuals and classified each effect as an outlier if its residual exceeded the bounds of ±1.96. Five effect sizes from four studies exceeded these bounds, resulting in a significant effect of nature with an estimated mean effect size of SMD = 0.486 (SE = 0.067; 95% CI[0.35, 0.62]), $t(54.9) = 7.23$, $p < .001$ and a prediction interval of [-0.541, 1.514].

Third, we investigated potential influential cases by applying several influence diagnostics. We estimated DFBETAS and Cook's Distances and coded studies with DFBETAS greater than one as potentially influential. Furthermore, we plotted Cook's distance and visually identified studies with large Cook's distance values. Based on these criteria, 24 effect sizes from 17 studies were removed, resulting in a significant effect of nature on self-reported pain with an estimated mean effect size of SMD = 0.447 (SE = 0.052; 95% CI[0.34, 0.55]), $t(37.5) = 8.63$, $p < .001$ and a prediction interval of [-0.277, 1.171].

Fourth, we excluded two studies that measured changes in spontaneous chronic pain during nature exposure[32,33]. Unlike the other included studies, which investigated

pain induced by medical or experimental procedures, these studies focused on spontaneously occurring pain directly associated with chronic conditions. Thus, we sought to determine whether their exclusion altered the main results. The exclusion of both studies resulted in 94 remaining effects from 60 studies, which were included in the multilevel model. The model demonstrated a significant effect of nature with an estimated mean effect size of SMD = 0.502 (SE = 0.082; 95% CI[0.34, 0.67]), $t(57.3) = 6.11$, $p < .001$ and a prediction interval of [-0.767, 1.771].

In summary, excluding outliers, influential cases, and studies investigating spontaneous chronic pain, yielded smaller but statistically significant effects of nature exposure on self-reported pain. Applying a stringent exclusion criterion led to the removal of 23 studies, resulting in an SMD of 0.484. Similarly, excluding 17 influential studies yielded an SMD of 0.447. As shown in Supplementary Table 3 and Supplementary Figure 2, both approaches notably reduced the effect size dispersion, narrowed the prediction intervals, and substantially decreased between-study heterogeneity. Specifically, the between-study heterogeneity dropped from 67.4% in the full model to 15.2% and 13.8% in the models excluding potential outliers and influential cases, respectively. These results suggest that a considerable portion of the observed heterogeneity was attributable to atypical studies, most of which reported high-to-very-high effect sizes. Crucially, excluding these studies, whether based on outlier or influential diagnostics, did not substantially alter the estimated mean effect. Across all sensitivity analyses, the effect remained statistically significant and of moderate magnitude. This indicates that while the original heterogeneity was high, the core effect was not an artifact of outlier-driven results. Instead, the consistency of the effect across sensitivity analyses supports the robustness and generalizability of the findings, even in the context of a heterogeneous study pool.

Certainty of evidence. Using the GRADE framework, we rated the overall certainty of evidence as low. Two domains — risk of bias and study inconsistency — were judged as serious limitations, leading to downgrading by two levels. Regarding risk of bias, the proportion of information from studies at high risk was sufficient to affect the interpretation of results. Particularly, we identified a key limitation in the outcome measurement domain (D4), arising from the inherently experiential and subjective nature of pain, which makes it susceptible to potential bias. However, because self-reported pain is widely regarded as a reference standard in pain assessment, this limitation is not specific to the current evidence base and is inherent across the broader field of pain research. High heterogeneity further led us to downgrade for inconsistency. However, we did not assign the maximum downgrade, as sensitivity analyses suggested that much of the heterogeneity was driven by outliers and influential studies, whose exclusion did not meaningfully alter the estimated mean effect. The remaining domains — indirectness, imprecision, and publication bias — were not considered serious limitations. While publication bias could not be ruled out, we did not downgrade certainty, as GRADE allows only the ratings “undetected” or “strongly suspected”, and the available evidence did not support the latter. Although the funnel plot showed asymmetry, 33 out of 96 effects were non-significant, and 6 significant effects pointed in the opposite direction, suggesting a pattern more consistent with heterogeneity and small-study effects rather than selective reporting. In sum, high risk of bias and inconsistency represent the main limitations, underscoring the need for more rigorous primary studies. Notably, low or very low certainty ratings are not unique to this research domain: over half of healthcare-related systematic reviews report low or very low certainty ratings[34], reflecting a broader methodological

challenge in evidence synthesis in healthcare research that naturally also affects the present research.

Association between moderator variables. To assess the interrelationships among moderator variables, we calculated Cohen's ω[35] for both preregistered and exploratory moderators: context, interactivity, immersiveness, type of control, design, type of outcome, purity and overall level of bias. The results are reported in Supplementary Table 4. Following established conventions[35], we interpret effects as small (<0.3), moderate (0.3-0.5), and large (>0.5). Of the 28 pairwise associations, 15 (53.6%) were small, 7 (25%) moderate, and 6 (21.4%) large in magnitude. A large association was observed between study design and context (ω = 0.81), reflecting that most experimental studies employed within-participant designs. In contrast, medical studies predominantly used between-participant or pre-post control group designs. Type of outcome showed a large association with study context (ω = 0.61) and study design (ω = 0.58), mirroring that all studies using threshold or tolerance measures were conducted in experimental settings and used within-participant designs. Purity was also strongly associated with immersiveness (ω = 0.51) and interactivity (ω = 0.63). This pattern reflects that confounded interventions often engaged two or more sensory modalities, typically involving active-attending or active-manipulation interventions. This is consistent with our inclusion criteria, which made it unlikely for unimodal interventions to be confounded by non-natural stimuli. The most substantial overlap was between interactivity and immersiveness (ω = 0.92). Interventions involving three sensory modalities were almost exclusively classified as active-manipulation, whereas unimodal interventions were predominantly categorized as passive-attending. Given the strong association between both variables, it is likely that the significant effect of immersiveness reflects, at least in part, variance shared with interactivity. Thus, while interactivity did not independently moderate effect sizes, it may still contribute to pain modulation in interaction with other features.

Supplementary Table 1. Studies excluded after full-text examination and reasons for exclusion.

Study	Code	Reason of exclusion
Araujo-Duran et al., 2023[36]	Insufficient intervention and control	The VR intervention included multiple programs, many of which may have not contained natural elements. The control condition included 2D presentations of nature.
Birrenbach et al., 2022[37]	Insufficient control	Participants reported their subjective pain levels both before and after a VR nature intervention. Thus, the study utilized a pre-post design but did not include a control group or condition.
Burrai et al., 2023[38]	Insufficient data	Descriptive statistics required for calculating the SMD were not provided.
Candela et al., 2023[39]	Insufficient control	Participants reported their subjective pain levels both before and after a VR nature intervention. Thus, the study utilized a pre-post design but did not include a control group or condition.
Chaze et al., 2022[40]	Several	The intervention included multiple environments, many of which contained non-natural elements. Additionally, pain was assessed using an observational tool, and the data provided were insufficient for calculating the SMD. Finally, there was no suitable control group or condition.
De Ramòn et al., 2023[41]	No access	No access to the manuscript.
Frere et al., 2001[42]	Insufficient data	Descriptive statistics required for calculating the SMD were not provided.
Glennon et al., 2018[43]	Insufficient control	Some participants in the control group were also exposed to nature stimuli.
Hoffman et al., 2006[44]	Insufficient data	Descriptive statistics required for calculating the SMD were not provided.
Hughes et al., 2019[45]	Insufficient control	Both the intervention and control conditions depicted natural scenes.
Kelleher et al., 2022[46]	Insufficient control	Participants reported their subjective pain levels before, during, and after a VR nature intervention. Thus, the study utilized a pre-post design but did not include a control group or condition.
Laghlam et al., 2021[47]	Insufficient control	The effects of viewing nature were compared to the administration of an analgesic.
Lohr & Pearson-Mims, 2000[48]	Insufficient data	Descriptive statistics required for calculating the SMD were not provided.
Mladenovic & Djordjevic, 2021[49]	Insufficient control	Participants underwent dental treatment, with the first half conducted using standard treatment and the second half incorporating VR nature as a distraction. There was no counterbalancing of the conditions.
Mühlberger et al., 2007[50]	Insufficient control	Both the VR intervention and control condition depicted natural scenes. The primary aim of the study was to compare the effects of nature stimuli administered via VR versus non-VR methods.
Park et al., 2008[51]	Insufficient data	Descriptive statistics required for calculating the SMD were not provided.
Park et al., 2009[52]	Insufficient data	Descriptive statistics required for calculating the SMD were not provided.
Patterson et al., 2006[53]	Insufficient data	The authors provided percentage change scores for worst pain from initial thermal pain to subsequent thermal pain. It was not possible to calculate the SMD based on the provided data.
Schneider et al., 2004[54]	Several	The authors used a composite measure as the dependent variable, which encompassed several constructs (e.g., pain, fatigue, nausea, concentration, etc.). SE or SD were not provided. Additionally, the VR scenarios included various environments without nature content, such as a museum experience and solving a mystery.
Ulrich, 1984[55]	Insufficient outcome	The outcome measure was the number and dose of administered analgesics. We rated it as too dissimilar from the outcomes used in the remaining studies.
Vincent et al., 2010[56]	Insufficient data	The authors did not provide information on the number of participants allocated to each group. Additionally, it was unclear whether the provided statistics represent SD or SE.
Wichrowski et al., 2021[57]	Insufficient outcome	The outcome measure used was a subjective report labelled "pain controlled". We rated it as too dissimilar from the outcomes used in the remaining studies.
Zhao et al., 2023[58]	Insufficient intervention	It is unclear which additional non-natural content was included in the VR intervention.

Note: SD = standard deviation; SE = standard error; SMD = standardized mean difference.

Supplementary Table 2. Studies included in the multilevel meta-analysis identified through the systematic literature review, including complete coding of variables.

Reference	Effect	Des.	Context	N	Pain<br>*Description*	*Measure*	Nature<br>*Description*	*Pure*[1]	*Immers-iveness*[2]	*Interactive*[3]	Control<br>*Description*	*Match*[4]	Data
Miller et al., 1992[59]	-	PPC	Medical	17	Burn wound dressing	NRS (no pain – excruciating pain)	Scenes of oceans, deserts, flowers, waterfalls and wildlife; shown on TV; music.	No	2	Passive-attending	TAU	No	Text
Lembo et al., 1998[60]	Auditory	B	Medical	24	Flexible sigmoidoscopy	VAS (faint pain – severely intense pain)	Sounds of ocean shore.	Yes	1	Passive-attending	TAU	No	Fig. 2
	Audio-visual	B	Medical	+13	Flexible sigmoidoscopy	VAS (faint pain – severely intense pain)	Scenes of ocean shore; shown on HMD; sounds of ocean shore.	Yes	2	Passive-attending	TAU	No	Fig. 2
Tse et al., 2002a[61]	Threshold	W	Exper-imental	46	Ischemic pain	Threshold (time until faint pain)	Scenes of nature (not specified); shown on HMD.	Yes	1	Passive-attending	Black screen	No	Tab. 1
	Tolerance	W	Exper-imental	-	Ischemic pain	Tolerance (time until intolerable pain)	Scenes of nature (not specified); shown on HMD.	Yes	1	Passive-attending	Black screen	No	Tab. 1
Tse et al., 2002b[62]	Threshold	W	Exper-imental	72	Ischemic pain	Threshold (time until faint pain)	Scenes of nature (such as mountains and waterfalls); shown on HMD.	Yes	1	Passive-attending	Black screen	No	Tab. 1
	Tolerance	W	Exper-imental	-	Ischemic pain	Tolerance (time until intolerable pain)	Scenes of nature (such as mountains and waterfalls); shown on HMD.	Yes	1	Passive-attending	Black screen	No	Tab. 1
Diette et al., 2003[16]	-	B	Medical	80	Flexible bronchoscopy	NRS (excellent control – poor control)	Scene of a stream in a spring meadow; shown on photographic mural; nature sounds.	Yes	2	Passive-attending	TAU	No	Tab. 4
Hoffman et al., 2004[63]	-	W	Exper-imental	8	Thermal pain	GRS (no pain – worst pain)	Scene of an icy virtual canyon including virtual objects (e.g., snowmen); shown on HMD; accompanied by integrated sound effects; interaction by throwing snowballs at objects (SnowWorld).	No	3	Active-manipulation	Fixation cross	No	Tab. 1
Lee et al., 2004[64]	Audio-visual	B	Medical	105	Colonoscopy	VAS (no pain – very painful)	Scenic views (not specified); shown on HMD; classical music.	No	2	Passive-attending	TAU	No	Tab. 3

	Visual	B	Medical	+52	Colonoscopy	VAS (no pain – very painful)	Scenic views (not specified); shown on HMD.	Yes	1	Passive-attending	TAU	No	Tab. 3
Hoffman et al., 2007[65]	-	W	Exper-imental	9	Thermal pain	GRS (no pain – worst pain)	Scene of an icy virtual canyon including virtual objects (e.g., snowmen); shown on HMD; integrated sound effects; interaction by throwing snowballs at objects (SnowWorld).	No	3	Active-manipulation	Fixation cross	No	Tab. 1
Furman et al., 2009[66]	Movie	W	Medical	38	Periodontal scaling and root planing	VAS (no pain – worst pain)	VR of a virtual botanical garden; shown on HMD; interaction by controlling movement and gaze of avatar through VR.	No	2	Active-navigation	Movie	Yes	Tab. 3
	Sham	W	Medical	-	Periodontal scaling and root planing	VAS (no pain – worst pain)	VR of a virtual botanical garden; shown on HMD; interaction by controlling movement and gaze of avatar through VR.	No	2	Active-navigation	Sham – HMD, no content	No	Tab. 3
Nikolajsen et al., 2009[67]	-	B	Medical	37	Placement of a femoral nerve block catheter	NRS (not indicated)	Scenes of nature (not specified); shown on 2D monitor; nature sounds and music.	No	2	Passive-attending	TAU	No	Fig. 2 & Text
Lechtzin et al., 2010[68]	TAU	B	Medical	81	Bone marrow aspiration biopsy	VAS (no pain – worst pain)	Scene of a stream in a spring meadow; shown on photographic mural; nature sounds.	Yes	2	Passive-attending	TAU	No	Text
	Urban	B	Medical	+39	Bone marrow aspiration biopsy	VAS (no pain – worst pain)	Scene of a stream in a spring meadow; shown on photographic mural; nature sounds.	Yes	2	Passive-attending	Scene of city; urban sounds	Yes	Text
Maani et al., 2011[69]	-	W	Medical	12	Burn wound dressing	GRS (no pain – worst pain)	Scene of an icy virtual canyon including virtual objects (e.g., snowmen); shown on HMD; integrated sound effects; interaction by throwing snowballs at objects (Snowworld).	No	3	Active-manipulation	TAU	No	Tab. 1
Garza-Villareal et al., 2012[70]	Rain	W	Exper-imental	48	Thermal pain	VAS (pain intensity; 0 – 100)	Sounds of rain.	Yes	1	Passive-attending	Pink noise	Yes	Fig. 2
	Water	W	Exper-imental	-	Thermal pain	VAS (pain intensity; 0 – 100)	Sounds of water.	Yes	1	Passive-attending	Pink noise	Yes	Fig. 2
de Tommaso et al., 2013[71]	Chronic	W	Exper-imental	16	Laser-evoked potentials	VAS (absence of pain – maximum pain)	VR of virtual hospital waiting room including large window with sea view; shown on HMD.	No	1	Active-attending	VR of standard hospital waiting room	Yes	Tab. 1

										without natural elements.		
	Healthy	W	Exper-imental	16	Laser-evoked potentials	VAS (absence of pain – maximum pain)	VR of virtual hospital waiting room including large window with sea view; shown on HMD.	No	1	Active-attending	VR of standard hospital waiting room without natural elements.	Yes	Tab. 1
Saadatmand et al., 2015[72]	-	PPC	Medical	60	Mechanical ventilation support	VAS (no pain – pain as bad as it can be)	Sounds of birds, rain, water (rivers, streams, waterfalls) or a forest walk.	Yes	1	Passive-attending	Sham – headphones no sound	No	Tab. 3
Ebrahimi et al., 2017[73]	2D	B	Medical	40	Burn wound dressing	VAS (no pain – intolerable pain)	Scene of waterfall; shown on 2D monitor; sounds of waterfall.	Yes	2	Passive-attending	NA	NA	Tab. 3
	VR	B	Medical	+20	Burn wound dressing	VAS (no pain – intolerable pain)	Scene of waterfall; shown on HMD; sounds of waterfall.	Yes	2	Active-attending	NA	NA	Tab. 3
McSherry et al., 2017[74]	-	PPC	Medical	30	Wound care	NRS (no pain – worst possible pain)	Scene of an icy virtual canyon including virtual objects (e.g., snowmen); shown on HMD; integrated sound effects; interaction by throwing snowballs at objects (Snowworld).	No	3	Active-manipulation	TAU	No	Tab. 3
Yildirim et al., 2017[75]	Affirmation	B	Medical	70	Chemotherapy infusion	VAS (no pain – worst pain)	Sounds of ocean and birds.	Yes	1	Passive-attending	Audio recording of affirmative sentences.	Yes	Pers. Com.
	TAU	B	Medical	+35	Chemotherapy infusion	VAS (no pain – worst pain)	Sounds of ocean and birds.	Yes	1	Passive-attending	TAU	No	Pers. Com.
Emami et al., 2018[76]	-	B	Medical	80	Cancer-related pain	VAS (low – high level of pain)	Scene of a lush hill with vegetation; viewed through window.	Yes	1	Passive-attending	Scene of urban area including street; viewed from window.	Yes	Tab. 2
Nielsen et al., 2018[77]	Music	B	Medical	126	Urological, gynecological, or orthopedic surgery	VAS (measuring pain; not further indicated)	Scenes of nature with green vegetation; shown on 2D monitor.	Yes	1	Passive-attending	Music: Soft instrumental music.	Yes	Tab. 3
	TAU	B	Medical	+41	Urological, gynecological, or orthopedic surgery	VAS (measuring pain; not further indicated)	Scenes of nature with green vegetation; shown on 2D monitor.	Yes	1	Passive-attending	TAU	No	Tab. 3

Schmidt et al., 2018[78]	-	W	Exper-imental	28	Electrical pain	VAS (not painful – unbearably painful)	Scenes of nature (e.g., fallen leaves); shown on 2D monitor.	Yes	1	Passive-attending	Black screen	No	Tab. 1
Soltani et al., 2018[79]	-	W	Medical	39	Range of motion exercises (burn wound patients)	GRS (no pain – worst pain)	Scene of an icy virtual canyon including virtual objects (e.g., snowmen); shown on HMD; integrated sound effects; interaction by throwing snowballs at objects (Snowworld).	No	3	Active-manipulation	TAU	No	Text
Tanja-Dijkstra et al., 2018[80]	Exp1 Active	B	Exper-imental	57	Thermal pain	NRS (no pain – pain as bad as can be)	VR of virtual coastal environment (e.g., sea, beach, field areas); shown on HMD; interaction by exploration using controller.	Yes	2	Active-navigation	Sham – HMD: no content.	No	Tab. 1
	Exp1 Passive	B	Exper-imental	+28	Thermal pain	NRS (no pain – pain as bad as can be)	VR of virtual coastal environment (e.g., sea, beach, field areas); shown on HMD.	Yes	1	Active-attending	Sham – HMD: no content.	No	Tab. 1
	Exp2 Control	B	Medical	48	Thermal pain	NRS (no pain – pain as bad as can be)	VR of virtual coastal environment (e.g., sea, beach, field areas); shown on HMD; interaction by exploration using controller.	Yes	2	Active-navigation	Sham – HMD: no content.	No	Tab. 5
	Exp2 Urban	B	Medical	+22	Dental treatment	NRS (no pain – pain as bad as can be)	VR of virtual coastal environment (e.g., sea, beach, field areas); shown on HMD; interaction by exploration using controller.	Yes	2	Active-navigation	VR of virtual urban environment shown on HMD; exploration using controller.	Yes	Tab. 5
Farzaneh et al., 2019[81]	Sham	PPC	Medical	38	Caesarean section	VAS (measuring pain; not further indicated)	Sounds of birds, rain, water or a jungle walk.	Yes	1	Passive-attending	Sham – Headphones : no audio.	No	Tab. 1
	TAU	PPC	Medical	+19	Caesarean section	VAS (measuring pain; not further indicated)	Sounds of birds, rain, water or a jungle walk.	Yes	1	Passive-attending	TAU	No	Tab. 1
Mohammad & Ahmad, 2019[32]	-	PPC	Medical	80	Spontaneous chronic pain	VAS (no pain – worst pain possible pain)	VR of underwater scene including animals or VR of beach; shown on HMD; natural sounds, music, and guided meditation.	No	3	Active-manipulation	TAU	No	Tab. 1, 2 & Text

Colloca et al., 2020[82]	Threshold	W	Exper-imental	49	Thermal pain	Threshold (time until minimally painful)	VR of underwater scene including animals; shown on HMD; natural sounds and relaxing music.	No	2	Active-navigation	VR of onstage opera experience; shown on HMD; music.	Yes	Tab. 1
	Tolerance	W	Exper-imental	-	Thermal pain	Tolerance (time until pain no longer endurable)	VR of underwater scene including animals; shown on HMD; natural sounds and relaxing music.	No	2	Active-navigation	VR of onstage opera experience; shown on HMD; music.	Yes	Tab. 1
Czub & Bagrij, 2020[83]	Tactile	W	Exper-imental	42	Thermal pain	Tolerance (time for hand kept in water)	VR of meadow and mountains; shown on HMD; interaction by working on virtual n-back task.	No	2	Active-manipulation	VR of virtual avatar; shown on HMD; interaction through tactile distraction device while working on n-back task.	Yes	Tab. 1
	TAU	W	Exper-imental	-	Thermal pain	Tolerance (time for hand kept in water)	VR of meadow and mountains; shown on HMD; interaction by working on virtual n-back task.	No	2	Active-manipulation	Black screen	No	Tab. 1
Lier et al., 2020[84]	Active	W	Exper-imental	29	Electrical pain	NRS (no pain – unbearable pain)	VR of boat ride on river; shown on HMD; nature sounds; interaction by shooting targets using keyboard.	No	3	Active-manipulation	Black screen	No	Tab. 1
	Passive	W	Exper-imental	-	Electrical pain	NRS (no pain – unbearable pain)	VR of boat ride on river; shown on HMD; nature sounds.	Yes	2	Active-attending	Black screen	No	Tab. 1
Araújo et al., 2021[85]	-	W	Medical	17	Wound dressing	VAS (no pain – worst pain imaginable)	VR of beaches, rural areas, and national parks; shown on HMD; nature sounds.	Yes	2	Active-navigation	TAU	No	Tab. 2
Basak et al., 2021[86]	Illusion	B	Medical	60	Intramuscular benzathine penicillin injection	VAS (no pain – most severe pain)	VR of submarine view; shown on HMD.	Yes	1	NA	Cards depicting optical illusions; participants had to indicate what is depicted on card.	Yes	Tab. 2

	TAU	B	Medical	+31	Intramuscular benzathine penicillin injection	VAS (no pain – most severe pain)	VR of submarine view; shown on HMD.	Yes	1	NA	TAU	No	Tab. 2
Cakir & Evirgen, 2021[87]	-	B	Medical	60	Colonoscopy	VAS (no pain – most severe pain)	VR of beach walk; shown on mobile phone placed in cardboard goggles; wave sounds and music.	No	2	Active-attending	TAU	No	Tab. 2
Deo et al., 2021[88]	-	B	Medical	40	Hysteroscopy	NRS (no pain – worst imaginable pain)	VR of virtual rainforest and lake including wildlife; shown on HMD; calming narration.	No	2	Active-attending	TAU	No	Tab. 2
Dings et al., 2021[89]	2D	B	Medical	104	Vasectomy	VAS (no pain – worst imaginable pain)	Scenes of nature (coastlines and the sea); shown on 2D video glasses.	Yes	1	Passive-attending	TAU	No	Tab. 2
	VR	B	Medical	+37	Vasectomy	VAS (no pain – worst imaginable pain)	Scenes of nature (north pole, dolphins underwater, safari); shown on HMD.	No	1	Active-attending	TAU	No	Tab. 2
Karaman & Taşdemir, 2021[90]	-	B	Medical	60	Fine needle aspiration breast biopsy	VAS (no pain – worst possible pain)	VR of beach walk; shown on mobile phone placed in cardboard goggles; wave sounds and music.	No	2	Active-attending	TAU	No	Tab. 2
Li et a., 2021[91]	2D Threshold	W	Exper-imental	24	Electrical pain	Threshold (time until slightest pain)	Scenes of nature (participant selected greenspace near home); shown on 2D monitor.	Yes	1	Passive-attending	Black screen	No	Text
	2D Tolerance	W	Exper-imental	-	Electrical pain	Tolerance (time until pain intolerable)	Scenes of nature (participant-selected greenspace near home); shown on 2D monitor.	Yes	1	Passive-attending	Black screen	No	Text
	Green Threshold	W	Exper-imental	-	Electrical pain	Threshold (time until slightest pain)	Real-World (in-situ) exposure to participant-selected greenspace.	Yes	4	Active-manipulation	Black screen	No	Text
	Green Tolerance	W	Exper-imental	-	Electrical pain	Tolerance (time until pain intolerable)	Real-World (in-situ) exposure to participant-selected greenspace.	Yes	4	Active-manipulation	Black screen	No	Text
Łuczak et al., 2021[92]	-	B	Medical	103	Rigid cystoscopy	NRS (no pain – unbearable pain)	VR of waterfall; shown on HMD; sounds of waterfall.	Yes	2	Active-attending	TAU	No	Tab. 2
Melcer et al., 2021[93]	-	B	Medical	60	Amniocentesis	VAS (no pain – severe pain)	VR of rolling hills, sailboat, tropical beach, desert landscape, undersea world; shown on HMD.	Yes	1	NA	TAU	No	Text
Momenyan et al., 2021[94]	-	PPC	Medical	48	Contractions during labor	NRS (no pain – worst pain)	VR of beach and peaceful landscape; shown on HMD; nature sounds.	Yes	2	Active-attending	TAU	No	Tab. 1 & 2

Verzwyvelt et al., 2021[95]	Green	PPC	Medical	33	Intravenous chemotherapy	NRS (no pain – excruciating/ unbearable pain)	Views of rooftop garden and mural depicting nature and abstract shapes.	No	1	Active-manipulation	TAU	No	Tab. 2
	VR	PPC	Medical	-	Intravenous chemotherapy	NRS (no pain – excruciating/ unbearable pain)	VR of natural environments (e.g., beaches, ocean); shown on HMD; interaction by exploration through movement.	Yes	2	Active-navigation	TAU	No	Tab. 2
Fouks et al., 2022[96]	-	B	Medical	82	Hysteroscopy	NRS (measuring pain; not further indicated)	VR of diving in a lagoon; shown on HMD; relaxing auditory stimuli and music.	No	3	Active-attending	TAU	No	Tab. 3 and text
Genc et al., 2022[18]	Stress ball	PPC	Medical	64	Transrectal prostate biopsy	VAS (no pain – most severe pain)	VR of nature video scenes (not specified); shown on HMD.	Yes	1	Active-attending	Squeeze stress ball and count to five (for 10 minutes)	Yes	Tab. 2
	TAU	PPC	Medical	+32	Transrectal prostate biopsy	VAS (no pain – most severe pain)	VR of nature video scenes (not specified); shown on HMD.	Yes	1	Active-attending	TAU	No	Tab. 2
Ketsuwan et al., 2022[97]		B	Medical	270	Colonoscopy	VAS (no pain – extreme pain)	VR of mountain range, sky, forest and falling snow; shown on HMD; relaxing music.	No	2	Active-attending	TAU	No	Tab. 2
Liu et al., 2022[98]	-	B	Medical	117	Colonoscopy	VAS (measuring pain; not further indicated)	VR of tropical islands and forests; shown on HMD; music.	No	2	NA	Sham - HMD: no content	No	Text, Fig. 1, Supp.
Perdue et al., 2022[99]	-	B	Medical	59	Intravenous catheter insertion	VAS (measuring pain; not further indicated)	VR of virtual underwater world including animals (e.g., coral reefs, lagoons); shown on HMD; interaction with animals possible.	Yes	3	Active-manipulation	Sham – HMD: no content	No	Tab. 1
Yesilot et al., 2022[100]	-	PPC	Medical	110	Laporoscopic surgery	NRS (no pain – unbearable pain)	VR of mountains, seas, and forests; shown on mobile phone placed in cardboard goggles; relaxing music.	No	2	NA	TAU	No	Tab. 2
Abbasnia et al., 2023[101]	Education	PPC	Medical	98	Laporoscopic surgery	VAS (measuring pain; not further indicated)	VR of nature, space, and ocean; shown on HMD.	Yes	1	Active-attending	VR movie showing animation-simulated film with pre-operative education.	Yes	Tab. 2
	TAU	PPC	Medical	+47	Laporoscopic surgery	VAS (measuring	VR of nature, space, and ocean; shown on HMD.	Yes	1	Active-attending	TAU	No	Tab. 2

						pain; not further indicated)						
Bi et al., 2023[102]	-	PPC	Exper-imental	32	Thermal pain	NRS (no pain – worst pain imaginable)	Two VR scenarios of deep ocean or seashore scene including animals; shown on HMD; nature sounds; guided respiration exercise (in scenario 2).	No	3	Active-manipulation	Control (not specified)	NA	Pers. Com.
Cakir & Evirgen, 2023[103]	Stress ball	B	Medical	60	Colonoscopy	VAS (absence of pain – most severe pain)	VR of beach walk; shown on mobile phone placed in cardboard goggles; wave sounds and music.	No	2	Active-attending	Squeeze stress ball and count to five (for 10 minutes)	Yes	Tab. 2
	Music	B	Medical	+30	Colonoscopy	VAS (absence of pain – most severe pain)	VR of beach walk; shown on mobile phone placed in cardboard goggles; wave sounds and music.	No	2	Active-attending	Exposure to classical turkish music via headphones	Yes	Tab. 2
	TAU	B	Medical	+30	Colonoscopy	VAS (absence of pain – most severe pain)	VR of beach walk; shown on mobile phone placed in cardboard goggles; wave sounds and music.	No	2	Active-attending	TAU	No	Tab. 2
Demirci et al., 2023[5]	-	PPC	Medical	50	Lower limb orthopedic surgery	NRS (no pain – worst pain possible)	Documentary of animal life; shown on HMD; narration and music.	No	2	Passive-attending	Music: Participant-selected preferred music.	Yes	Tab. 5
Girishan Prabhu et al., 2023[104]	2D	PPC	Medical	18	Knee arthroplasty	VAS (no pain – highest perceived pain)	Natural virtual scene including sky, waves, sun, and vegetation; shown on 2D display; interaction through HRV biofeedback controlling level of fog.	No	2	Active-manipulation	TAU	No	Tab. 3
	VR	PPC	Medical	+8	Knee arthroplasty	VAS (no pain – highest perceived pain)	VR virtual scene including sky, waves, sun, and vegetation; shown on HMD; interaction through HRV biofeedback controlling level of fog.	No	2	Active-manipulation	TAU	No	Tab. 3
Gullo et al., 2023[105]	-	B	Medical	100	Peripheral endovascular interventions (EVI) under local anesthesia	NRS (no pain – highest pain)	VR of virtual underwater world; shown on HMD; interaction through breathing; hypnosis narrative.	No	2	Active-manipulation	TAU	No	Tab. 2
Li et al., 2023[106]	Exp1 2D	W	Exper-imental	31	Thermal pain	NRS (no pain – pain as bad as it could be)	Two scenarios (deep ocean or seashore) including animals; shown on 2D monitor; nature	No	3	Active-manipulation	Fixation Cross	No	Fig. 2

							sounds; interaction through scene exploration (scene 1) or guided respiration exercise (in scenario 2).						
	Exp1 VR	W	Exper-imental	-	Thermal pain	NRS (no pain – pain as bad as it could be)	Two scenarios (deep ocean or seashore) including animals; shown on HMD; nature sounds; interaction through scene exploration (scene 1) or guided respiration exercise (in scenario 2).	No	3	Active-manipulation	Fixation Cross	No	Fig. 2
	Exp2 2D	W	Exper-imental	24	Thermal pain	NRS (no pain – pain as bad as it could be)	Two scenarios (deep ocean or seashore) including animals; shown on 2D monitor; nature sounds; interaction through scene exploration (scene 1) or guided respiration exercise (in scenario 2).	No	3	Active-manipulation	Fixation Cross	No	Fig. 1, Supp.
	Exp2 VR	W	Exper-imental	-	Thermal pain	NRS (no pain – pain as bad as it could be)	Two scenarios (deep ocean or seashore) including animals; shown on HMD; nature sounds; interaction through scene exploration (scene 1) or guided respiration exercise (in scenario 2).	No	3	Active-manipulation	Fixation Cross	No	Fig. 1, Supp.
Morris et al., 2023[107]	2D	PPC	Medical	48	Hospitalized traumatic injury patients	NRS (no pain – worst possible pain)	Virtual scene of coral reef with aquatic wildlife; shown on 2D monitor; calming music.	No	2	Active-navigation	Sham – HMD: no content.	No	Fig. 2
	VR	PPC	Medical	-	Hospitalized traumatic injury patients	NRS (no pain – worst possible pain)	Virtual scene of coral reef with aquatic wildlife; shown on HMD; calming music.	No	2	Active-navigation	Sham – HMD: no content.	No	Fig. 2
Pelazas-Hernández et al., 2023[108]	-	B	Medical	154	Hysteroscopy	VAS (no pain – worst pain imaginable)	Virtual scene of a vale with an open night-sky; shown on HMD; sound effects and music; interaction through controller to connect star patterns.	No	3	Active-manipulation	TAU	No	Tab. 2
Perenic et al., 2023[109]	-	B	Medical	153	Prostate biopsy	VAS (no pain – worst pain)	Virtual scenes including three landscapes (snow world, forest, garden); shown on HMD; relaxing music.	No	2	Active-attending	TAU	No	Tab. 2
Sewell et al., 2023[110]	-	PPC	Medical	83	Hysteroscopy	NRS (no pain – worst pain)	Nature scene of walk-through zen garden with changing seasons; shown on HMD; relaxing music and guided breathing exercise.	No	3	Active-manipulation	TAU	No	Tab. 1 & 3
Singh et al., 2023[111]	-	B	Medical	99	Various painful procedures	NRS (measuring	VR of virtual beach; shown on HMD.	Yes	1	NA	TAU	No	Tab. 2

						pain; not further indicated)							
Sooriyaghandan et al., 2023[112]	-	PPC	Medical	80	Flexible bronchoscopy	VAS (no pain – unbearable pain)	Nature scenes (not specified); shown on HMD; soothing music.	No	2	Active-attending	TAU	No	Pers. Com.
Yamashita et al., 2023[113]	Game	W	Exper-imental	46	Electrical pain	Threshold (level of electric current perceived as painful)	Nature scenes (e.g., rivers, animals); shown on HMD; music.	No	2	Passive-attending	Video game where participants use controller to shoot targets.	Yes	Pers. Com.
	TAU	W	Exper-imental	-	Electrical pain	Threshold (level of electric current perceived as painful)	Nature scenes (e.g., rivers, animals); shown on HMD; music.	No	2	Passive-attending	No distraction (not specified)	No	Pers. Com.
Yilmaz et al., 2023[114]	-	PPC	Medical	44	Colonoscopy	VAS (no pain – unbearable pain)	Nature scenes (not specified); shown on HMD; music.	No	2	Active-attending	TAU	No	Tab. 2
Girishan Prabhu et al., 2024[115]	-	PPC	Medical	58	Breast biopsy	VAS (measuring pain; not further indicated)	Scene of beach environment; shown on HMD; sounds of sea waves and wind.	Yes	2	Active-attending	TAU	No	Tab. 1
Gungormus et al., 2024[33]	-	PPC	Medical	42	Spontaneous chronic pain	NRS (no pain – worst pain imaginable)	Multisensory stimulation with biotic (plants) and abiotic (stones, soil) natural materials; interaction through transplanting of plants to pots.	Yes	4	Active-manipulation	Multisensory stimulation with synthetic imitations of materials used in experimental group.	Yes	Tab. 2

Note: *Note:* 2D = two-dimensional display; B = between-participant; Des. = Design type; Fig. = Figure; GRS = graphical rating scale; HMD = head-mounted display; N = sample size ('+' signs indicate additional participants included in studies with multiple effect sizes); NRS = numerical rating scale; Pers. Com. = Personal communication with study author; PPC = pre-post control; Tab. = Table; TAU = treatment as usual; VAS = visual analogue scale; VR = virtual reality; W = within-participant;
1: Pure indicates whether there were additional, non-natural confounding elements present during the nature intervention (e.g., narration, music, gamification, etc.);
2: Immersiveness indicates how many sensory modalities were targeted from 1 to 3 modalities & additionally category 4 = "real-world" exposure;
3: Interactive indicates the degree the intervention was interactive: Passive-attending = no interaction; Active-attending = freely look around in 360-degree image; Active-navigation = navigate through environment; Active-manipulation = manipulate objects in environments;
4: Match indicates whether the comparators were relatively matched or non-matched.

Supplementary Table 3. Characteristics of the full model and models excluding outliers, influential cases, and studies assessing spontaneous chronic pain.

Model	SMD	95% CI	p	95% PI	k effects removed
Full model	0.53	0.36 - 0.70	<.001	-0.78 - 1.85	-
Outliers removed[1]	0.48	0.40 - 0.57	<.001	0.07 - 0.90	42
Outliers removed[2]	0.49	0.35 - 0.62	<.001	-0.54 - 1.51	5
Influential cases removed[3]	0.45	0.34 - 0.55	<.001	-0.28 - 1.17	24
Spontaneous chronic pain studies removed	0.50	0.34 - 0.67	<.001	-0.77 - 1.77	2

Note: [1]Outliers based on CI, [2]Outliers based on externally studentized residuals, [3]Influential cases based on Cook's Distance and DFBETA values; CI = confidence interval; k = number of effect sizes removed; PI = prediction interval; SMD = standardized mean difference.

Supplementary Table 4. Pairwise associations (Cohen's ω) among preregistered and exploratory moderator variables. Values reflect the degree of association between categorical moderators.

	Interactive	Immersive	Control	Design	Outcome	Purity	Bias
Context	0.26	0.29	0.08	0.81	0.61	0.04	0.48
Interactive		0.92	0.22	0.41	0.28	0.51	0.21
Immersive			0.25	0.31	0.32	0.63	0.33
Control				0.11	0.06	0.03	0.34
Design					0.58	0.19	0.38
Outcome						0.06	0.23
Purity							0.21

Note: Cohen's ω values: <0.3 = small, 0.3-.0.5 = moderate, >0.5 = large.

Supplementary Figure 1 brief title: Forest plot depicting the effect of nature interventions on self-reported pain using within-study aggregated estimates.

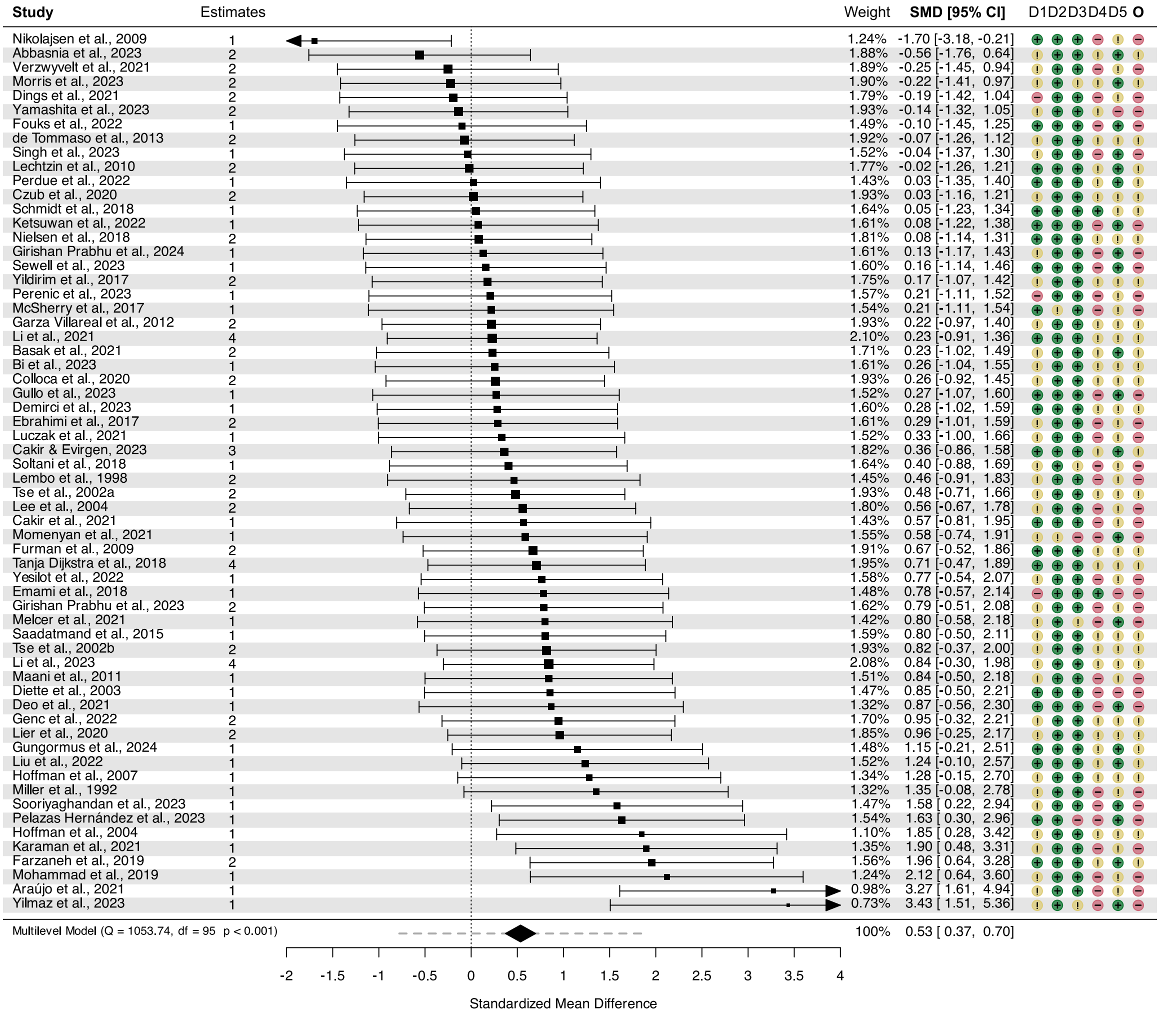

Supplementary Figure 1. Forest plot depicting the effect of nature interventions on self-reported pain using within-study aggregated estimates. Individual effect sizes (standardized mean differences; SMD) are shown as squares, with error bars indicating their 95% confidence intervals. Higher and positive values represent reductions in self-reported pain during nature interventions. The number of estimates refers to the effect sizes extracted per study. The estimated mean effect size (SMD = .535, p = .000000029) and its 95% confidence interval are shown as a diamond, based on k = 96 effects from n = 62 studies. The grey dotted line indicates the prediction interval for the estimated mean effect size [-0.78, 1.85]. The estimated mean effect is based on a three-level intercept-only meta-analysis with robust variance estimation (two-sided). The annotations D1-D5 correspond to the domains of the Cochrane Risk of Bias assessment: D1 = Randomization process; D2 = Deviations from intended intervention; D3 = Missing outcome data; D4 = Measurement of the outcome; D5 = Selection of the reported result; O = Overall bias. Note that the risk of bias was assessed at the study level and is color-coded: green represents low concern, yellow represents some concern, and red represents high concern. The confidence intervals per study are relatively large due to within-study aggregation.

Supplementary Figure 2 brief title: Density plots of effect size distributions comparing models across subsets of studies.

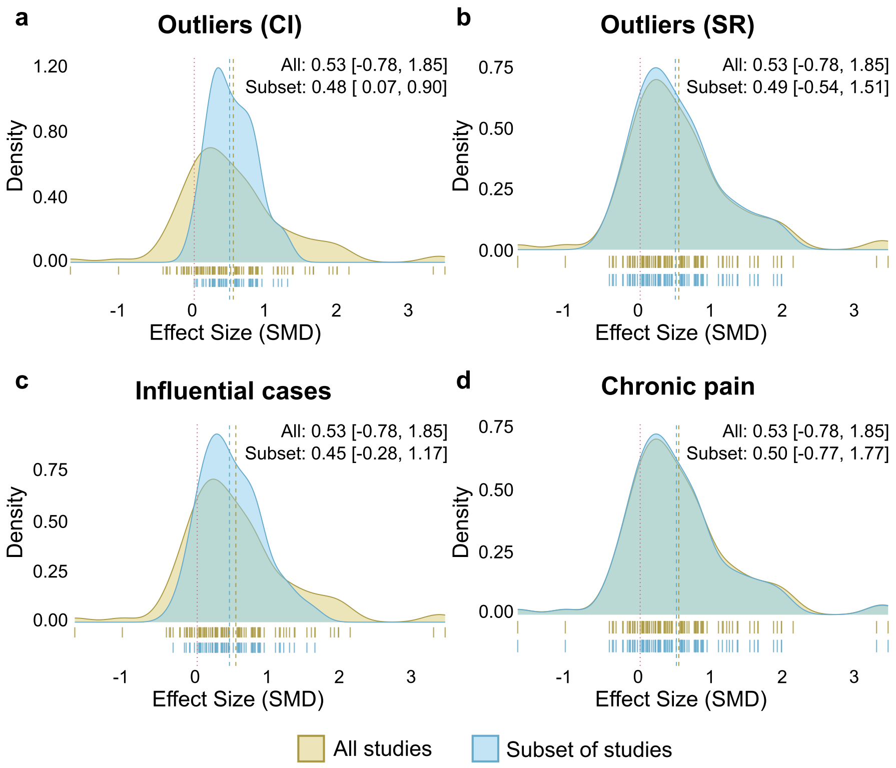

Supplementary Figure 2. Density plots of effect size distributions comparing models including all studies (yellow) versus subsets excluding specific studies (blue; k = number of effects; n = number of studies). Panels show exclusions based on: (a) outliers identified by non-overlapping 95% confidence intervals (CI; k = 54, n = 39); (b) outliers identified using studentized residuals (SR; k = 91, n = 58); (c) influential cases identified via Cook's distance and DFBETAs (k = 72, n = 45); (d) studies investigating spontaneously occurring chronic pain (k = 94, n = 60). Vertical tick marks below each density curve indicate individual effect sizes for each set. Reported values are standardized mean differences (SMD) with 95% prediction intervals in square brackets. Dashed vertical lines indicate SMD estimates based on all studies (yellow) or the corresponding subset (blue). The SMDs are based on a three-level intercept-only meta-analysis with robust variance estimation (two-sided).

Supplementary References

1. Schünemann, H., Brożek, J., Guyatt, G. & Oxman, A. *GRADE Handbook for Grading Quality of Evidence and Strength of Recommendations*. (The GRADE Working Group, 2013).
2. Higgins, J. P. T. *et al. Cochrane Handbook for Systematic Reviews of Interventions*. (Cochrane, 2024).
3. McMaster University and Evidence Prime. GRADEpro GDT: GRADEpro Guideline Development Tool. McMaster University and Evidence Prime (2025).
4. McGrath, S., Zhao, X., Steele, R. & Benedetti, A. *Estmeansd: Estimating the Sample Mean and Standard Deviation from Commonly Reported Quantiles in Meta-Analysis*. (2023).
5. Demirci, H. *et al.* Watching a movie or listening to music is effective in managing perioperative anxiety and pain: A randomised controlled trial. *Knee Surg Sports Traumatol Arthrosc* **31**, 6069–6079 (2023).
6. Wan, X., Wang, W., Liu, J. & Tong, T. Estimating the sample mean and standard deviation from the sample size, median, range and/or interquartile range. *BMC Med Res Methodol* **14**, 135 (2014).
7. Hedges, L. V. Distribution theory for glass's estimator of effect size and related estimators. *J Educ Stat* **6**, 107–128 (1981).
8. Cousineau, D. Approximating the distribution of Cohen's d_p in within-subject designs. *Quant Method Psychol* **16**, 418–421 (2020).
9. Becker, B. J. Synthesizing standardized mean-change measures. *Brit J Math & Statis* **41**, 257–278 (1988).
10. Morris, S. B. Estimating effect sizes from pretest-posttest-control group designs. *Organ Res Methods* **11**, 364–386 (2008).
11. Alghadir, A., Anwer, S., Iqbal, A. & Iqbal, Z. Test-retest reliability, validity, and minimum detectable change of visual analog, numerical rating, and verbal rating scales for measurement of osteoarthritic knee pain. *JPR* **Volume 11**, 851–856 (2018).
12. Brunelli, C. *et al.* Comparison of numerical and verbal rating scales to measure pain exacerbations in patients with chronic cancer pain. *Health Qual Life Outcomes* **8**, 42 (2010).
13. Gallagher, E. J., Bijur, P. E., Latimer, C. & Silver, W. Reliability and validity of a visual analog scale for acute abdominal pain in the ED. *Am J Emerg Med* **20**, 287–290 (2002).
14. Sendlbeck, M., Araujo, E. G., Schett, G. & Englbrecht, M. Psychometric properties of three single-item pain scales in patients with rheumatoid arthritis seen during routine clinical care: A comparative perspective on construct validity, reproducibility and internal responsiveness. *RMD Open* **1**, e000140 (2015).
15. Sindhu, B. S., Shechtman, O. & Tuckey, L. Validity, reliability, and responsiveness of a digital version of the visual analog scale. *J Hand Ther* **24**, 356–364 (2011).
16. Diette, G. B., Lechtzin, N., Haponik, E., Devrotes, A. & Rubin, H. R. Distraction therapy with nature sights and sounds reduces pain during flexible bronchoscopy: A complementary approach to routine analgesia. *Chest* **123**, 941–948 (2003).
17. Borenstein, M., Hedges, L. V., Higgins, J. & Rothstein, H. R. *Introduction to Meta-Analysis*. (Wiley, Hoboken, NJ, 2021).
18. Genc, H., Korkmaz, M. & Akkurt, A. The effect of virtual reality glasses and stress balls on pain and vital findings during transrectal prostate biopsy: A randomized controlled trial. *J Perianesth Nurs* **37**, 344–350 (2022).

19. Assink, M. & Wibbelink, C. J. M. Fitting three-level meta-analytic models in R: A step-by-step tutorial. *Tutor Quant Methods Psychol* **12**, 154–174 (2016).
20. Cheung, M. W.-L. Modeling dependent effect sizes with three-level meta-analyses: A structural equation modeling approach. *Psychol Methods* **19**, 211–229 (2014).
21. Van Den Noortgate, W., López-López, J. A., Marín-Martínez, F. & Sánchez-Meca, J. Three-level meta-analysis of dependent effect sizes. *Behav Res Methods* **45**, 576–594 (2013).
22. Hox, J. J., Moerbeek, M. & Schoot, R. van de. *Multilevel Analysis: Techniques and Applications*. (Routledge, Taylor & Francis Group, New York London, 2018).
23. Pustejovsky, J. E. & Tipton, E. Meta-analysis with robust variance estimation: Expanding the range of working models. *Prev Sci* **23**, 425–438 (2022).
24. Bhalang, K., Sigurdsson, A., Slade, G. D. & Maixner, W. Associations among four modalities of experimental pain in women. *J Pain* **6**, 604–611 (2005).
25. Defrin, R., Shramm, L. & Eli, I. Gender role expectations of pain is associated with pain tolerance limit but not with pain threshold. *Pain* **145**, 230–236 (2009).
26. Lue, Y. -J., Wang, H. -H., Cheng, K. -I., Chen, C. -H. & Lu, Y. -M. Thermal pain tolerance and pain rating in normal subjects: Gender and age effects. *Eur J Pain* **22**, 1035–1042 (2018).
27. Viechtbauer, W. & Cheung, M. W.-L. Outlier and influence diagnostics for meta-analysis. *Res Synth Method* **1**, 112–125 (2010).
28. Rodgers, M. A. & Pustejovsky, J. E. Evaluating meta-analytic methods to detect selective reporting in the presence of dependent effect sizes. *Psychol Methods* **26**, 141–160 (2021).
29. Pustejovsky, J. E. *clubSandwich: Cluster-Robust (Sandwich) Variance Estimators with Small-Sample Corrections*. (2023).
30. Harrer, M., Cuijpers, P., Furukawa, T. A. & Ebert, D. D. *Doing Meta-Analysis with R: A Hands-On Guide*. (Chapman and Hall/CRC, Boca Raton, 2021). doi:10.1201/9781003107347.
31. Viechtbauer, W. Conducting meta-analyses in R with the metafor package. *J Stat Soft* **36**, 1–48 (2010).
32. Mohammad, E. & Ahmad, M. Virtual reality as a distraction technique for pain and anxiety among patients with breast cancer: A randomized control trial. *Pall Supp Care* **17**, 29–34 (2019).
33. Gungormus, D. B., Fernández-Martín, M., Ortigosa-Luque, M. E. & Pérez-Mármol, J. M. Effects of nature-based multisensory stimulation on pain mechanisms in women with fibromyalgia syndrome: A randomized double-blind placebo-controlled trial. *Pain Manag Nurs* **25**, 46–55 (2024).
34. Howick, J. *et al.* Most healthcare interventions tested in Cochrane Reviews are not effective according to high quality evidence: A systematic review and meta-analysis. *J Clin Epidemiol* **148**, 160–169 (2022).
35. Cohen, J. *Statistical Power Analysis for the Behavioral Sciences*. (Lawrence Erlbaum, Hillsdale, 1988).
36. Araujo-Duran, J. *et al.* Virtual reality distraction for reducing acute postoperative pain after hip arthroplasty: A randomized trial. *Anesth Analg* **138**, 751–759 (2023).
37. Birrenbach, T. *et al.* Virtual reality for pain relief in the emergency room (VIPER) – A prospective, interventional feasibility study. *BMC Emerg Med* **22**, 113 (2022).
38. Burrai, F., Ortu, S., Marinucci, M., De Marinis, M. G. & Piredda, M.

Effectiveness of immersive virtual reality in people with cancer undergoing antiblastic therapy: A randomized controlled trial. *Semin Oncol Nurs* **39**, 151470 (2023).
39. Candela, L. *et al.* The use of a virtual reality device (HypnoVR) during extracorporeal shockwave lithotripsy for treatment of urinary stones: Initial results of a clinical protocol. *Urology* **175**, 13–17 (2023).
40. Chaze, F. *et al.* Virtual reality and well-being in older adults: Results from a pilot implementation of virtual reality in long-term care. *J Rehabil Assist Technol Eng* **9**, 205566832110723 (2022).
41. De Ramón, L. A. S., Martínez, A. F. F., Carricondo, A. R. G., Gálvez, F. E. & Rodríguez, R. A. Effect of virtual reality and music therapy on anxiety and perioperative pain in surgical extraction of impacted third molars. *J Am Dent Assoc* **154**, 206–214 (2023).
42. Frere, C. L., Crout, R., Yorty, J. & McNeil, D. W. Effects of audiovisual distraction during dental prophylaxis. *J Am Dent Assoc* **132**, 1031–1038 (2001).
43. Glennon, C. *et al.* Use of virtual reality to distract from pain and anxiety. *Oncol Nurs Forum* **45**, 545–552 (2018).
44. Hoffman, H. G. *et al.* Virtual reality helmet display quality influences the magnitude of virtual reality analgesia. *J Pain* **7**, 843–850 (2006).
45. Hughes, S. W., Zhao, H., Auvinet, E. J. & Strutton, P. H. Attenuation of capsaicin-induced ongoing pain and secondary hyperalgesia during exposure to an immersive virtual reality environment. *Pain Rep* **4**, e790 (2019).
46. Kelleher, S. A. *et al.* Virtual reality for improving pain and pain-related symptoms in patients with advanced stage colorectal cancer: A pilot trial to test feasibility and acceptability. *Palliat Support Care* **20**, 471–481 (2022).
47. Laghlam, D. *et al.* Virtual reality vs. Kalinox® for management of pain in intensive care unit after cardiac surgery: A randomized study. *Ann Intensive Care* **11**, 74 (2021).
48. Lohr, V. I. & Pearson-Mims, C. H. Physical discomfort may be reduced in the presence of interior plants. *Horttechnology* **10**, 53–58 (2000).
49. Mladenovic, R. & Djordjevic, F. Effectiveness of virtual reality as a distraction on anxiety and pain during impacted mandibular third molar surgery under local anesthesia. *J Stomatol Oral Maxillofac Surg* **122**, e15–e20 (2021).
50. Mühlberger, A., Wieser, M. J., Kenntner-Mabiala, R., Pauli, P. & Wiederhold, B. K. Pain modulation during drives through cold and hot virtual environments. *Cyberpsychol Behav* **10**, 516–522 (2007).
51. Park, S.-H. & Mattson, R. H. Effects of flowering and foliage plants in hospital rooms on patients recovering from abdominal surgery. *Horttechnology* **18**, 563–568 (2008).
52. Park, S.-H. & Mattson, R. H. Ornamental indoor plants in hospital rooms enhanced health outcomes of patients recovering from surgery. *J Altern Complement Med* **15**, 975–980 (2009).
53. Patterson, D. R., Hoffman, H. G., Palacios, A. G. & Jensen, M. J. Analgesic effects of posthypnotic suggestions and virtual reality distraction on thermal pain. *J Abnorm Psychol* **115**, 834–841 (2006).
54. Schneider, S. M., Prince-Paul, M., Allen, M. J., Silverman, P. & Talaba, D. Virtual reality as a distraction intervention for women receiving chemotherapy. *Oncol Nurs Forum* **31**, 81–88 (2004).
55. Ulrich, R. S. View through a window may influence recovery from surgery. *Science* **224**, 420–421 (1984).
56. Vincent, E., Battisto, D., Grimes, L. & McCubbin, J. The effects of nature

images on pain in a simulated hospital patient room. *HERD* **3**, 42–55 (2010).
57. Wichrowski, M. J., Corcoran, J. R., Haas, F., Sweeney, G. & Mcgee, A. Effects of biophilic nature imagery on indexes of satisfaction in medically complex physical rehabilitation patients: An exploratory study. *HERD* **14**, 288–304 (2021).
58. Zhao, X., Ma, J., Fang, R. & Wei, C. Effect of virtual reality distraction on satisfaction and tolerability in patients undergoing in-office KTP laser procedure. *J Voice* **S0892-1997**, 00160–1 (2023).
59. Miller, A. C., Hickman, L. C. & Lemasters, G. K. A distraction technique for control of burn pain. *J Burn Care Rehabil* **13**, 576–580 (1992).
60. Lembo, T. *et al.* Audio and visual stimulation reduces patient discomfort during screening flexible sigmoidoscopy. *Am J Gastroenterol* **93**, 1113–1116 (1998).
61. Tse, M. M. Y., Ng, J. K. F., Chung, J. W. Y. & Wong, T. K. S. The effect of visual stimuli on pain threshold and tolerance. *J Clin Nurs* **11**, 462–469 (2002).
62. Tse, M. M. Y., Ng, J. K. F., Chung, J. W. Y. & Wong, T. K. S. The effect of visual stimulation via the eyeglass display and the perception of pain. *Cyberpsychol Behav* **5**, 65–75 (2002).
63. Hoffman, H. G. *et al.* Modulation of thermal pain-related brain activity with virtual reality: Evidence from fMRI. *NeuroReport* **15**, 1245–1248 (2004).
64. Lee, D. *et al.* Can visual distraction decrease the dose of patient-controlled sedation required during colonoscopy? A prospective randomized controlled trial. *Endoscopy* **36**, 197–201 (2004).
65. Hoffman, H. G. *et al.* The analgesic effects of opioids and immersive virtual reality distraction: Evidence from subjective and functional brain imaging assessments. *Anesth Analg* **105**, 1776–1783 (2007).
66. Furman, E. *et al.* Virtual reality distraction for pain control during periodontal scaling and root planing procedures. *J Am Dent Assoc* **140**, 1508–1516 (2009).
67. Nikolajsen, L., Lyndgaard, K., Schriver, N. B. & Moller, J. F. Does audiovisual stimulation with music and nature sights (MuViCure) reduce pain and discomfort during placement of a femoral nerve block? *J Perianesth Nurs* **24**, 14–18 (2009).
68. Lechtzin, N. *et al.* A randomized trial of nature scenery and sounds versus urban scenery and sounds to reduce pain in adults undergoing bone marrow aspirate and biopsy. *J Altern Complement Med* **16**, 965–972 (2010).
69. Maani, C. V. *et al.* Virtual reality pain control during burn wound debridement of combat-related burn injuries using robot-like arm mounted VR goggles. *J Trauma* **71**, S125-130 (2011).
70. Garza Villarreal, E. A., Brattico, E., Vase, L., Østergaard, L. & Vuust, P. Superior analgesic effect of an active distraction versus pleasant unfamiliar sounds and music: The influence of emotion and cognitive style. *PLoS ONE* **7**, e29397 (2012).
71. de Tommaso, M. *et al.* Virtual visual effect of hospital waiting room on pain modulation in healthy subjects and patients with chronic migraine. *Pain Res Treat* **2013**, 515730 (2013).
72. Saadatmand, V. *et al.* Effects of natural sounds on pain: A randomized controlled trial with patients receiving mechanical ventilation support. *Pain Manag Nurs* **16**, 483–492 (2015).
73. Ebrahimi, H., Namdar, H., Ghahramanpour, M., Ghafourifard, M. & Musavi, S. Effect of virtual reality method and multimedia system on burn patients' pain during dressing. *J Clin Anal Med* **8**, 485–489 (2017).
74. McSherry, T. *et al.* Randomized, crossover study of immersive virtual reality to decrease opioid use during painful wound care procedures in adults. *J Burn Care*

Res **39**, 278–285 (2017).
75. Yildirim, M. *et al.* Symptom management: The effects of self-affirmation on chemotherapy-related symptoms. *Clin J Oncol Nurs* **21**, E15–E22 (2017).
76. Emami, E., Amini, R. & Motalebi, G. The effect of nature as positive distractibility on the healing process of patients with cancer in therapeutic settings. *Complement Ther Clin Pract* **32**, 70–73 (2018).
77. Nielsen, E., Wåhlin, I. & Frisman, G. H. Evaluating pictures of nature and soft music on anxiety and well-being during elective surgery. *Open Nurs J* **12**, 58–66 (2018).
78. Schmidt, K., Gamer, M., Forkmann, K. & Bingel, U. Pain affects visual orientation: An eye-tracking study. *J Pain* **19**, 135–145 (2018).
79. Soltani, M. *et al.* Virtual reality analgesia for burn joint flexibility: A randomized controlled trial. *Rehabil Psychol* **63**, 487–494 (2018).
80. Tanja-Dijkstra, K. *et al.* The soothing sea: A virtual coastal walk can reduce experienced and recollected pain. *Environ Behav* **50**, 599–625 (2018).
81. Farzaneh, M. *et al.* Comparative effect of nature-based sounds intervention and headphones intervention on pain severity after cesarean section: A prospective double-blind randomized trial. *Anesth Pain Med* **9**, e67835 (2019).
82. Colloca, L. *et al.* Virtual reality: Physiological and behavioral mechanisms to increase individual pain tolerance limits. *Pain* **161**, 2010–2021 (2020).
83. Czub, M. & Bagrij, A. Tactile and visual virtual reality attention distraction from pain in cold pressor test. *Pol Psychol Bull* **51**, 315–323 (2020).
84. Lier, E. J., Oosterman, J. M., Assmann, R., de Vries, M. & van Goor, H. The effect of virtual reality on evoked potentials following painful electrical stimuli and subjective pain. *Sci Rep* **10**, 9067 (2020).
85. Araújo, T. M., da Silva, A. S. J., Brandão, M. G. S. A., Barros, L. M. & Veras, V. S. Virtual reality in pain relief during chronic wound dressing change. *Rev esc enferm USP* **55**, e20200513 (2021).
86. Basak, T., Demirtas, A. & Yorubulut, S. M. Virtual reality and distraction cards to reduce pain during intramuscular benzathine penicillin injection procedure in adults: A randomized controlled trial. *J Adv Nurs* **77**, 2511–2518 (2021).
87. Cakir, S. K. & Evirgen, S. The effect of virtual reality on pain and anxiety during colonoscopy: A randomized controlled trial. *Turk J Gastroenterol* **32**, 451–457 (2021).
88. Deo, N. *et al.* Virtual reality for acute pain in outpatient hysteroscopy: A randomised controlled trial. *BJOG* **128**, 87–95 (2021).
89. Dings, S. J. M., van Stralen, K. J., Struben, V. M. D. & Noordzij, M. A. Pain and anxiety during vasectomies while distracting patients with video glasses or virtual reality glasses. *BJU Int* **128**, 561–567 (2021).
90. Karaman, D. & Taşdemir, N. The effect of using virtual reality during breast biopsy on pain and anxiety: A randomized controlled trial. *J Perianesth Nurs* **36**, 702–705 (2021).
91. Li, H., Zhang, X., Bi, S., Cao, Y. & Zhang, G. Can residential greenspace exposure improve pain experience? A comparison between physical visit and image viewing. *Healthcare* **9**, 918 (2021).
92. Łuczak, M. *et al.* Influence of virtual reality devices on pain and anxiety in patients undergoing cystoscopy performed under local anaesthesia. *J Pers Med* **11**, 1214 (2021).
93. Melcer, Y. *et al.* Analgesic efficacy of virtual reality for acute pain in amniocentesis: A randomized controlled trial. *Eur J Obstet Gynecol Reprod Biol* **261**, 134–138 (2021).

94. Momenyan, N., Safei, A. A. & Hantoushzadeh, S. Immersive virtual reality analgesia in un-medicated laboring women (during stage 1 and 2): A randomized controlled trial. *Clin Exp Obstet Gynecol* **48**, 110–116 (2021).
95. Verzwyvelt, A. L., McNamara, A., Xu, X. & Stubbins, R. Effects of virtual reality v. biophilic environments on pain and distress in oncology patients: A case-crossover pilot study. *Sci Rep* **11**, 20196 (2021).
96. Fouks, Y. *et al.* A virtual reality system for pain and anxiety management during outpatient hysteroscopy: A randomized control trial. *Eur J Pain* **26**, 600–609 (2022).
97. Ketsuwan, C. *et al.* Prospective randomized controlled trial to evaluate effectiveness of virtual reality to decrease anxiety in office-based flexible cystoscopy patients. *World J Urol* **40**, 2575–2581 (2022).
98. Liu, Q. *et al.* Implementation of virtual reality technology to decrease patients' pain and nervousness during colonoscopies: A prospective randomised controlled single-blinded trial. *Clin Med* **22**, 237–240 (2022).
99. Perdue, M. J., Umar, M. A., Walker, J. D. & Kubena, B. Immersive virtual reality for pain control and anxiolysis during IV blood draws in adults: A randomized controlled trial. *Mil Med* **188**, e2467–e2471 (2022).
100. Yesilot, S. B., Yeşilkuş, R. & Beyaz, F. Use of virtual reality for reducing pain and anxiety after laparoscopic sleeve gastrectomy: A randomized controlled trial. *Pain Manag Nurs* **23**, 826–831 (2022).
101. Abbasnia, F., Aghebati, N., Miri, H. H. & Etezadpour, M. Effects of patient education and distraction approaches using virtual reality on pre-operative anxiety and post-operative pain in patients undergoing laparoscopic cholecystectomy. *Pain Manag Nurs* **24**, 280–288 (2023).
102. Bi, Y. *et al.* Enhancing pain modulation: The efficacy of synchronous combination of virtual reality and transcutaneous electrical nerve stimulation. *Gen Psych* **36**, e101164 (2023).
103. Cakir, S. K. & Evirgen, S. Three distraction methods for pain reduction during colonoscopy: A randomized controlled trial evaluating the effects on pain and anxiety. *J Perianesth Nurs* **38**, e1–e7 (2023).
104. Girishan-Prabhu, V. G., Stanley, L., Morgan, R. & Shirley, B. Designing and developing a nature-based virtual reality with heart rate variability biofeedback for surgical anxiety and pain management: Evidence from total knee arthroplasty patients. *Aging Ment Health* **28**, 738–753 (2023).
105. Gullo, G. *et al.* Virtually augmented self-hypnosis in peripheral vascular intervention: A randomized controlled trial. *Cardiovasc Intervent Radiol* **46**, 786–793 (2023).
106. Li, J. *et al.* The analgesic effects and neural oscillatory mechanisms of virtual reality scenes based on distraction and mindfulness strategies in human volunteers. *Br J Anaesth* **131**, 1082–1092 (2023).
107. Morris, N. A. *et al.* Adjunctive virtual reality pain relief after traumatic injury: A proof-of-concept within-person randomized trial. *Pain* **164**, 2122–2129 (2023).
108. Pelazas-Hernández, J. A. *et al.* The effect of virtual reality on the reduction of pain in women with an indication for outpatient diagnostic hysteroscopy: A randomized controlled trial. *J Clin Med* **12**, 3645 (2023).
109. Perenic, E., Grember, E., Bassard, S. & Koutlidis, N. Impact of virtual reality on pain management in transrectal MRI-guided prostate biopsy. *Front Pain Res* **4**, 1156463 (2023).
110. Sewell, T., Fung, Y., Al-Kufaishi, A., Clifford, K. & Quinn, S. Does virtual reality technology reduce pain and anxiety during outpatient hysteroscopy? A

randomised controlled trial. *BJOG* **130**, 1466–1472 (2023).
111. Singh, N. *et al.* The use of immersive audiovisual distraction with virtual reality during pain procedures: A randomized controlled trial. *Pain Med* **24**, 1204–1206 (2023).
112. Sooriyaghandan, I. V. *et al.* Satisfaction and tolerability using virtual reality (VR) as adjunctive treatment during flexible bronchoscopy: A randomized control trial. *BMC Pulm Med* **23**, 10 (2023).
113. Yamashita, Y., Aijima, R. & Danjo, A. Clinical effects of different virtual reality presentation content on anxiety and pain: A randomized controlled trial. *Sci Rep* **13**, 20487 (2023).
114. Yılmaz, E. D. & Dinçer, N. Ü. The effects of virtual reality glasses on vital signs and anxiety in patients undergoing colonoscopy: A randomized controlled trial. *Gastroenterol Nurs* **46**, 318–328 (2023).
115. Girishan-Prabhu, V. G. *et al.* The impact of virtual reality on anxiety and pain during US-Guided breast biopsies: A randomized controlled clinical trial. *J Breast Imaging* **6**, 45–52 (2024).

PRISMA 2020 Checklist

Section and Topic	Item #	Checklist item	Location where item is reported
**TITLE**			
Title	1	Identify the report as a systematic review.	Title (p. 1), Abstract (p. 1), Introduction (p. 3), Discussion (p. 6), Methods (p. 10); Supplementary Information (p. 1, 2)
**ABSTRACT**			
Abstract	2	See the PRISMA 2020 for Abstracts checklist.	Due to abstract word limits, only the most critical items are summarized in the abstract. Full adherence to all items is detailed in the main manuscript.
**INTRODUCTION**			
Rationale	3	Describe the rationale for the review in the context of existing knowledge.	Introduction (p. 1-3)
Objectives	4	Provide an explicit statement of the objective(s) or question(s) the review addresses.	Introduction (p. 3)
**METHODS**			
Eligibility criteria	5	Specify the inclusion and exclusion criteria for the review and how studies were grouped for the syntheses.	Methods (p. 10-11); Supplementary Information (p. 2)
Information sources	6	Specify all databases, registers, websites, organisations, reference lists and other sources searched or consulted to identify studies. Specify the date when each source was last searched or consulted.	Methods (p. 11); Supplementary Information (p. 2)
Search strategy	7	Present the full search strategies for all databases, registers and websites, including any filters and limits used.	Supplementary Information (p. 2)
Selection process	8	Specify the methods used to decide whether a study met the inclusion criteria of the review, including how many reviewers screened each record and each report retrieved, whether they worked independently, and if applicable, details of automation tools used in the process.	Methods (p. 11)
Data collection process	9	Specify the methods used to collect data from reports, including how many reviewers collected data from each report, whether they worked independently, any processes for obtaining or confirming data from study investigators, and if applicable, details of automation tools used in the process.	Methods (p. 11-12)
Data items	10a	List and define all outcomes for which data were sought. Specify whether all results that were compatible with each outcome domain in each study were sought (e.g. for all measures, time points, analyses), and if not, the methods used to decide which results to collect.	Methods (p. 10)
	10b	List and define all other variables for which data were sought (e.g. participant and intervention characteristics, funding sources). Describe any assumptions made about any missing or unclear information.	Methods (p. 10-12); Supplementary Information (p. 2-3)
Study risk of bias assessment	11	Specify the methods used to assess risk of bias in the included studies, including details of the tool(s) used, how many reviewers assessed each study and whether they worked independently, and if applicable, details of automation tools used in the process.	Results (p. 4); Methods (p. 13)

Section and Topic	Item #	Checklist item	Location where item is reported
Effect measures	12	Specify for each outcome the effect measure(s) (e.g. risk ratio, mean difference) used in the synthesis or presentation of results.	Methods (p. 11-12); Supplementary Information (p. 3-4)
Synthesis methods	13a	Describe the processes used to decide which studies were eligible for each synthesis (e.g. tabulating the study intervention characteristics and comparing against the planned groups for each synthesis (item #5)).	Supplementary Information (p. 2)
	13b	Describe any methods required to prepare the data for presentation or synthesis, such as handling of missing summary statistics, or data conversions.	Methods (p. 11-12); Supplementary Information (p. 3)
	13c	Describe any methods used to tabulate or visually display results of individual studies and syntheses.	Results (p. 3, 5-6); Table 1; Supplementary Table 2; Figure 2-4; Supplementary Figure 1-2
	13d	Describe any methods used to synthesize results and provide a rationale for the choice(s). If meta-analysis was performed, describe the model(s), method(s) to identify the presence and extent of statistical heterogeneity, and software package(s) used.	Methods (p. 11-12); Supplementary Information (p. 3-4)
	13e	Describe any methods used to explore possible causes of heterogeneity among study results (e.g. subgroup analysis, meta-regression).	Methods (p. 11-12); Results (p. 5); Table 2; Supplementary Information (p. 5, 6)
	13f	Describe any sensitivity analyses conducted to assess robustness of the synthesized results.	Methods (p. 12); Results (p. 5); Supplementary Information (p. 5-6)
Reporting bias assessment	14	Describe any methods used to assess risk of bias due to missing results in a synthesis (arising from reporting biases).	Results (p. 6, p. 12); Figure 4; Methods (p. 12)
Certainty assessment	15	Describe any methods used to assess certainty (or confidence) in the body of evidence for an outcome.	Supplementary Information (p. 3)
**RESULTS**			
Study selection	16a	Describe the results of the search and selection process, from the number of records identified in the search to the number of studies included in the review, ideally using a flow diagram.	Results (p. 3); Figure 1
	16b	Cite studies that might appear to meet the inclusion criteria, but which were excluded, and explain why they were excluded.	Results (p. 3); Supplementary information (p. 2); Supplementary Table 1
Study characteristics	17	Cite each included study and present its characteristics.	Results (p. 3-6); Table 1; References; Supplementary Table 2; Supplementary References
Risk of bias in studies	18	Present assessments of risk of bias for each included study.	Figure 2; Supplementary Figure 1
Results of individual studies	19	For all outcomes, present, for each study: (a) summary statistics for each group (where appropriate) and (b) an effect estimate and its precision (e.g. confidence/credible interval), ideally using structured tables or plots.	Figure 2; Supplementary Figure 1; Accompanying dataset (nersep_data_update.csv)
Results of syntheses	20a	For each synthesis, briefly summarise the characteristics and risk of bias among contributing studies.	Discussion (p. 6-10); Figure 2; Supplementary Figure 1
	20b	Present results of all statistical syntheses conducted. If meta-analysis was done, present for each the summary estimate and its precision (e.g. confidence/credible interval) and measures of	Results (p. 5-6); Supplementary Information (p. 5-7)

Section and Topic	Item #	Checklist item	Location where item is reported
		statistical heterogeneity. If comparing groups, describe the direction of the effect.	
	20c	Present results of all investigations of possible causes of heterogeneity among study results.	Results (p. 5-6); Table 2; Figure 3; Supplementary Information (p. 5)
	20d	Present results of all sensitivity analyses conducted to assess the robustness of the synthesized results.	Results (p. 5); Supplementary Information (p. 5-6); Supplementary Table 3
Reporting biases	21	Present assessments of risk of bias due to missing results (arising from reporting biases) for each synthesis assessed.	Results (p. 6); Figure 4
Certainty of evidence	22	Present assessments of certainty (or confidence) in the body of evidence for each outcome assessed.	Supplementary Information (p. 6)
**DISCUSSION**			
Discussion	23a	Provide a general interpretation of the results in the context of other evidence.	Discussion (p. 6-10)
	23b	Discuss any limitations of the evidence included in the review.	Discussion (p. 6-10)
	23c	Discuss any limitations of the review processes used.	Discussion (p. 6-10)
	23d	Discuss implications of the results for practice, policy, and future research.	Discussion (p. 9-10)
**OTHER INFORMATION**			
Registration and protocol	24a	Provide registration information for the review, including register name and registration number, or state that the review was not registered.	Methods (p. 10)
	24b	Indicate where the review protocol can be accessed, or state that a protocol was not prepared.	No separate protocol was prepared beyond the registration on PROSPERO which outlines the key planned methods.
	24c	Describe and explain any amendments to information provided at registration or in the protocol.	No changes were made to registered information.
Support	25	Describe sources of financial or non-financial support for the review, and the role of the funders or sponsors in the review.	Acknowledgements (p. 21)
Competing interests	26	Declare any competing interests of review authors.	Competing Interest Statement (p. 21)
Availability of data, code and other materials	27	Report which of the following are publicly available and where they can be found: template data collection forms; data extracted from included studies; data used for all analyses; analytic code; any other materials used in the review.	Data extracted from included studies; Data used for all analyses; Analytic code – links reported in Data availability and Code availability section (p. 13)
