# Supplementary material for: Nature exposure reduces self-reported pain: a systematic review and meta-analysis
Source: Nat Ment Health. 2026 Jan 6;4(1):165–80. doi: 10.1038/s44220-025-00569-2 (PMC12789040; doi:10.1038/s44220-025-00569-2)
Supplement: Supplementary file 2 — Reporting Summary [file 44220_2025_569_MOESM2_ESM.pdf]

nature portfolio

Corresponding author(s): Claus Lamm

Last updated by author(s): Oct 10, 2025

Reporting Summary

Nature Portfolio wishes to improve the reproducibility of the work that we publish. This form provides structure for consistency and transparency in reporting. For further information on Nature Portfolio policies, see our Editorial Policies and the Editorial Policy Checklist.

Statistics

For all statistical analyses, confirm that the following items are present in the figure legend, table legend, main text, or Methods section.

n/a	Confirmed	
☐	☒	The exact sample size (*n*) for each experimental group/condition, given as a discrete number and unit of measurement
☐	☒	A statement on whether measurements were taken from distinct samples or whether the same sample was measured repeatedly
☐	☒	The statistical test(s) used AND whether they are one- or two-sided<br>*Only common tests should be described solely by name; describe more complex techniques in the Methods section.*
☐	☒	A description of all covariates tested
☐	☒	A description of any assumptions or corrections, such as tests of normality and adjustment for multiple comparisons
☐	☒	A full description of the statistical parameters including central tendency (e.g. means) or other basic estimates (e.g. regression coefficient) AND variation (e.g. standard deviation) or associated estimates of uncertainty (e.g. confidence intervals)
☐	☒	For null hypothesis testing, the test statistic (e.g. F, t, r) with confidence intervals, effect sizes, degrees of freedom and P value noted<br>*Give P values as exact values whenever suitable.*
☒	☐	For Bayesian analysis, information on the choice of priors and Markov chain Monte Carlo settings
☒	☐	For hierarchical and complex designs, identification of the appropriate level for tests and full reporting of outcomes
☒	☐	Estimates of effect sizes (e.g. Cohen's d, Pearson's r), indicating how they were calculated

Our web collection on statistics for biologists contains articles on many of the points above.

Software and code

Policy information about availability of computer code

Data collection

No custom or commercial software was used to collect data. Studies were identified through manual searches of online databases including PubMed, PsycINFO, Web of Science, and SCOPUS. When outcome data was not available in the text, tables, or accompanying raw data, we extracted data from figures using WebPlotDigitizer (version 5.2; https://automeris.io).

Extracted data were recorded and organized using Microsoft Excel - Microsoft Office 365 (2025; version 16.98)

The code used to analyse the data is publicly available under: https://osf.io/hmf4r/

Data analysis

Meta-analysis - including moderator and sensitivity analyses, as well as data visualization - was conducted using R (R Core Team, 2025; version 4.4.3), and the metafor (Viechtbauer, 2010; version 4.8.0), clubsandwich (Pustejovsky, 2024; version 0.5.11), and orchaRd package (Nakagawa et al., 2023; version 2.0). Furthermore, data were visualized using ggplot2 (Wickham, 2016; version 3.5.1).

Risk of Bias was assessed and coded using the RoB2 Excel tool: Sterne, J. A., Savović, J., Page, M. J., Elbers, R. G., Blencowe, N. S., Boutron, I., Cates, C.J., Cheng, H., Corbett, M.S., Eldridge, S.M., Emberson, J. R., Hernán, M.A., Hopewell, S., Hróbjartsson, A., Junqueira, D.R., Jüni, P., Kirkham, J.J., Lasserson, T., Tianjing, L., ... & Higgins, J. P. (2019). RoB 2: a revised tool for assessing risk of bias in randomised trials. BMJ, 366. https://doi.org/10.1136/bmj.l4898

For manuscripts utilizing custom algorithms or software that are central to the research but not yet described in published literature, software must be made available to editors and reviewers. We strongly encourage code deposition in a community repository (e.g. GitHub). See the Nature Portfolio guidelines for submitting code & software for further information.

Data

Policy information about availability of data

All manuscripts must include a data availability statement. This statement should provide the following information, where applicable:

- Accession codes, unique identifiers, or web links for publicly available datasets
- A description of any restrictions on data availability
- For clinical datasets or third party data, please ensure that the statement adheres to our policy

The extracted data — including means, standard deviations, sample sizes, study and effect IDs, and moderator coding — have been deposited on OSF and are accessible at https://osf.io/hmf4r/ . Additionally, the Source Data for all Figures and Tables are provided as a separate Source Data file.

Research involving human participants, their data, or biological material

Policy information about studies with human participants or human data. See also policy information about sex, gender (identity/presentation), and sexual orientation and race, ethnicity and racism.

Reporting on sex and gender	The studies included in this systematic review and meta-analysis involved both male and female participants. Data on sex/gender distribution was reported in 58 out of 62 studies. Among these, a total of 4,422 participants had reported sex/gender data: 1,989 male (45%) and 2,433 female (55%). Six studies included only male participants, and eleven studies included only female participants.<br>No analyses were conducted separately by sex/gender, and no sex- or gender-specific hypotheses were formulated in the systematic review and meta-analysis. Methods for determining sex/gender were not consistently reported across studies, which hindered a clear distinction between the two. The data shared on OSF do not include disaggregated sex/gender data, as this information was not consistently available across studies and individual-level data were not extracted.
Reporting on race, ethnicity, or other socially relevant groupings	The systematic review and meta-analysis did not include analyses based on socially constructed categorization variables such as ethnicity or education, as no hypotheses were specified for these factors. Furthermore only limited data was available in the primary studies, rendering meaningful synthesis challenging. Out of 62 studies 17 (27.4%) reported participant's educational background, and 10 (16.1%) reported ethnicitiy. However, the methods of classification were not consistent and often not clearly described.
Population characteristics	Population characteristics varied across studies, reflecting differences in design, settings, and participant demographics. Mean age was reported in 52 out of 62 studies and ranged from 20.33-70.94 years with a weighted arithmetic mean of 44.91 years.
Recruitment	We did not recruit participants for this study. All data were obtained from previously published studies.
Ethics oversight	We used secondary anonymized data from existing and published studies, which did not require additional ethical approval.

Note that full information on the approval of the study protocol must also be provided in the manuscript.

Field-specific reporting

Please select the one below that is the best fit for your research. If you are not sure, read the appropriate sections before making your selection.

☐ Life sciences ☒ Behavioural & social sciences ☐ Ecological, evolutionary & environmental sciences

For a reference copy of the document with all sections, see nature.com/documents/nr-reporting-summary-flat.pdf

Behavioural & social sciences study design

All studies must disclose on these points even when the disclosure is negative.

Study description	We conducted a systematic review and meta-analysis, including sensitivity and moderator (subgroup) analyses of studies investigating the effect of nature exposure on acute self-reported pain. All data represent secondary data and are quantitative.
Research sample	All included studies in the article were collected up until 31/01/2024. We included studies involving adult participants from healthy and clinical populations undergoing experimental or medical procedures typically experienced as painful. Studies had to feature at least one intervention that stimulated one or more sensory modalities (e.g., visual, auditory, tactile) primarily using natural stimuli and include some form of comparator (matched or non-matched). The unifying feature across all interventions was that nature-based sensory input was the central component of the intervention. Self-reported pain was required as an outcome, assessed using either scales (visual analogue (VAS), numerical rating (NRS), or graphical rating scales (GRS)) or measures of pain threshold or tolerance. We included studies with between-participant, within-participant, or pre-post control group designs. Eligible studies had to be published in English, peer-reviewed journals, and present original research (excluding opinion pieces, reviews, etc.). Studies were deemed ineligible if they employed inadequate interventions, comparators, outcomes, provided insufficient data, or were grey literature. As we used secondary data from existing research, the overall sample may not be representative.

Sampling strategy	As we used secondary data of existing and published studies, we did not predetermine sample sizes.
Data collection	We searched the extent of the literature up until January 31, 2024 (with no restrictions on earlier publication date) using four electronic databases: PsychINFO, PubMed, Web of Science (WOS), and SCOPUS. Search terms combined concepts related to nature interventions, pain outcomes, and exclusion criteria. The same terms were used across all four databases, with adjustments made for database-specific search rules. Additionally, we performed reference tracing through forward and backward tracking. Two reviewers (MOS and JPN) independently reviewed, coded (e.g., study characteristics, moderators, and risk of bias), and extracted data from all studies. Titles and abstracts were first screened against predfined inclusion criteria, followed by full-text examinations of eligible articles. Discrepancies and ambiguities were resolved through direct discussion between the reviewers.<br><br>Data were tabulated and organized using Microsoft Excel (see above for details).<br><br>The initial search resulted in 2,413 records. After removing 1,116 duplicates and 19 case reports, 1,278 records were screened. Of these 1,194 records were excluded based on title and abstract review. We then examined full-texts of the remaining records from which 23 were excluded for not meeting the inclusion criteria. From the final 62 included studies, we extracted 96 individual effect sizes.
Timing	Studies were identified through two separate searches: The initial search was completed on October 31, 2022, and the final search was completed on January 31, 2024.
Data exclusions	Studies were excluded if they did not meet the inclusion criteria. Eligible studies were required to: (1) include human adults from healthy or clinical populations, (2) involve procedures perceived as painful, (3) feature interventions that primarily stimulated one or more sensory modalities using natural stimuli, (4) include a comparator condition to these stimuli, (5) report self-reported pain as an outcome, (6) be published in English in a peer-reviewed journal, and (7) present original research. All studies not meeting these criteria were excluded. Additionally, 23 studies that passed the initial screening (title and abstract) were excluded after full-text examination. Reasons for exclusion varied and encompassed insufficient control conditions, inadequate data presentation, lack of access to full text, insufficient outcome measures, and inadequate interventions.
Non-participation	Of the 62 studies included in the meta-analysis 17 (27.4%) did not report whether any participants dropped out, 24 studies (38.7%) stated that no participants dropped out after randomization, and 21 studies (33.8%) reported participant dropouts after randomization, with an average dropout rate of 11%. Reported reasons for dropouts varied but commonly included technical issues, protocol violations, missing or poor-data quality, and side effects related to the interventions under investigation.
Randomization	Allocation of participants into experimental groups or conditions was randomized in 59 of the included studies and non-randomized in 3 studies.

Reporting for specific materials, systems and methods

We require information from authors about some types of materials, experimental systems and methods used in many studies. Here, indicate whether each material, system or method listed is relevant to your study. If you are not sure if a list item applies to your research, read the appropriate section before selecting a response.

Materials & experimental systems

n/a	Involved in the study
☒	☐ Antibodies
☒	☐ Eukaryotic cell lines
☒	☐ Palaeontology and archaeology
☒	☐ Animals and other organisms
☒	☐ Clinical data
☒	☐ Dual use research of concern
☒	☐ Plants

Methods

n/a	Involved in the study
☒	☐ ChIP-seq
☒	☐ Flow cytometry
☒	☐ MRI-based neuroimaging

Plants

Seed stocks	*Report on the source of all seed stocks or other plant material used. If applicable, state the seed stock centre and catalogue number. If plant specimens were collected from the field, describe the collection location, date and sampling procedures.*
Novel plant genotypes	*Describe the methods by which all novel plant genotypes were produced. This includes those generated by transgenic approaches, gene editing, chemical/radiation-based mutagenesis and hybridization. For transgenic lines, describe the transformation method, the number of independent lines analyzed and the generation upon which experiments were performed. For gene-edited lines, describe the editor used, the endogenous sequence targeted for editing, the targeting guide RNA sequence (if applicable) and how the editor was applied.*
Authentication	*Describe any authentication procedures for each seed stock used or novel genotype generated. Describe any experiments used to assess the effect of a mutation and, where applicable, how potential secondary effects (e.g. second site T-DNA insertions, mosiacism, off-target gene editing) were examined.*
